# Comparative efficacy of interventions to promote hand hygiene in hospital: systematic review and network meta-analysis

**DOI:** 10.1136/bmj.h3728

**Published:** 2015-07-28

**Authors:** Nantasit Luangasanatip, Maliwan Hongsuwan, Direk Limmathurotsakul, Yoel Lubell, Andie S Lee, Stephan Harbarth, Nicholas P J Day, Nicholas Graves, Ben S Cooper

**Affiliations:** 1 Mahidol-Oxford Tropical Medicine Research Unit, Faculty of Tropical Medicine, Mahidol University, Bangkok, Thailand; 2 School of Public Health, Queensland University of Technology, Brisbane, Australia; 3 Department of Tropical Hygiene, Faculty of Tropical Medicine, Mahidol University, Bangkok, Thailand; 4 Centre for Tropical Medicine and Global Health, Nuffield Department of Clinical Medicine, University of Oxford, Oxford, UK; 5 Infection Control Program, University of Geneva Hospitals and Faculty of Medicine, Geneva 1211, Switzerland; 6 Departments of Infectious Diseases and Microbiology, Royal Prince Alfred Hospital, Sydney 2050, Australia; 7 Institute of Health and Biomedical Innovation, Queensland University of Technology, Brisbane, Australia

## Abstract

**Objective** To evaluate the relative efficacy of the World Health Organization 2005 campaign (WHO-5) and other interventions to promote hand hygiene among healthcare workers in hospital settings and to summarize associated information on use of resources.

**Design** Systematic review and network meta-analysis.

**Data sources** Medline, Embase, CINAHL, NHS Economic Evaluation Database, NHS Centre for Reviews and Dissemination, Cochrane Library, and the EPOC register (December 2009 to February 2014); studies selected by the same search terms in previous systematic reviews (1980-2009).

**Review methods** Included studies were randomised controlled trials, non-randomised trials, controlled before-after trials, and interrupted time series studies implementing an intervention to improve compliance with hand hygiene among healthcare workers in hospital settings and measuring compliance or appropriate proxies that met predefined quality inclusion criteria. When studies had not used appropriate analytical methods, primary data were re-analysed. Random effects and network meta-analyses were performed on studies reporting directly observed compliance with hand hygiene when they were considered sufficiently homogeneous with regard to interventions and participants. Information on resources required for interventions was extracted and graded into three levels.

**Results** Of 3639 studies retrieved, 41 met the inclusion criteria (six randomised controlled trials, 32 interrupted time series, one non-randomised trial, and two controlled before-after studies). Meta-analysis of two randomised controlled trials showed the addition of goal setting to WHO-5 was associated with improved compliance (pooled odds ratio 1.35, 95% confidence interval 1.04 to 1.76; I^2^=81%). Of 22 pairwise comparisons from interrupted time series, 18 showed stepwise increases in compliance with hand hygiene, and all but four showed a trend for increasing compliance after the intervention. Network meta-analysis indicated considerable uncertainty in the relative effectiveness of interventions, but nonetheless provided evidence that WHO-5 is effective and that compliance can be further improved by adding interventions including goal setting, reward incentives, and accountability. Nineteen studies reported clinical outcomes; data from these were consistent with clinically important reductions in rates of infection resulting from improved hand hygiene for some but not all important hospital pathogens. Reported costs of interventions ranged from $225 to $4669 (£146-£3035; €204-€4229) per 1000 bed days.

**Conclusion** Promotion of hand hygiene with WHO-5 is effective at increasing compliance in healthcare workers. Addition of goal setting, reward incentives, and accountability strategies can lead to further improvements. Reporting of resources required for such interventions remains inadequate.

## Introduction

At any point in time more than 1.4 million patients around the world experience healthcare associated infections.^1^ ^2^ Such infections cause excess morbidity and are associated with increased mortality.^2^ ^3^ Direct contact between patients and healthcare workers who are transiently contaminated with nosocomial pathogens is believed to be the primary route of transmission for several organisms and can lead to patients becoming colonised or infected. Although hand hygiene is widely thought to be the most important activity for the prevention of nosocomial infections, a review of hand hygiene studies by the World Health Organization (WHO) found that baseline compliance with hand hygiene among healthcare workers was on average only 38.7% (range 5-89%).^4^

In 2005, the WHO World Alliance for Patient Safety launched a campaign, the First Global Patient Safety Challenge—“Clean Care is Safer Care”—aiming to improve hand hygiene in healthcare.^4^ This campaign (WHO-5) promotes a multimodal strategy consisting of five components: system change, training and education, observation and feedback, reminders in the hospital, and a hospital safety climate. More recently, additional strategies for improving hand hygiene have been evaluated, including those based on behavioural theory.

We assessed the relative effectiveness of WHO-5 and other strategies for improving compliance with hand hygiene in healthcare workers in hospital settings. Evaluation of the evidence for the effectiveness of different interventions is complicated by three factors: firstly, most evaluations of interventions to promote hand hygiene use non-randomised study designs, and in many cases the reported analysis is inappropriate or methodological quality is too low to allow meaningful conclusions to be drawn^5^ ^6^ ^7^ ^8^; secondly, there is wide variation between studies in the activities to promote hand hygiene used in the comparison group; thirdly, direct head-to-head comparisons of most interventions are lacking.^7^

We aimed to overcome these problems by restricting attention to randomised trials and high quality non-randomised studies, re-analysing data when necessary; explicitly accounting for activities to promote hand hygiene in the comparison group in each study; and using network meta-analysis to allow indirect comparison between interventions.

We also summarise information on changes in clinical and microbiological outcomes associated with interventions when this was reported. Information on resources used in different interventions is essential for those wanting to implement such interventions or evaluate their cost effectiveness.^9^ ^10^ An additional aim was therefore to document information on resources used in interventions to promote hand hygiene.

## Methods

We developed a protocol and used systematic methods to identify relevant studies, screen study eligibility, and assess study quality. This protocol was not registered. This review is reported according to the Preferred Reporting Items for Systematic Reviews and Meta-Analyses (PRISMA) guidelines.^11^

### Search strategy

We used a two stage search strategy. Firstly, we obtained all studies considered in two previous reviews (covering the period up to November 2009), including those that had been reported as failing to meet inclusion criteria.^5^ ^6^ Secondly, we extended the search from these studies from December 2009 to February 2014. We searched Medline, Embase, Cumulative Index to Nursing and Allied Health (CINAHL), Database of Abstracts of Reviews of Effects (DARE), National Health Service Economic Evaluation Database (NHS-EED), National Health Service Centre for Reviews and Dissemination (NHS-CRD) and British Nursing Index (BNI), Cochrane Library (Cochrane database of systematic reviews, Cochrane central register of controlled trials, Cochrane methodology register, Health Technology assessment database), Clinical Trial.gov, Current Clinical Control trial, Cochrane Effective Practice and Organisation of Care Group (EPOC) register, American College of Physicians journal, and reviews of evidence based medicine. Results were limited to peer reviewed publications. To validate previous search results we also repeated the electronic search for three earlier years (1980, 1995, and 2009). The complete search strategy is provided in appendix 1.

### Inclusion and exclusion

Studies were included if they met all the following initial criteria: they evaluated one or more interventions intended to improve hand hygiene compliance among healthcare workers in a hospital setting; they measured compliance with hand hygiene using opportunities with prespecified indications or using proxies linked to compliance (such as consumption of soap and alcohol hand rub); they were either randomised controlled trials, non-randomised trials, controlled before-after studies, or used an interrupted time series design.

We placed no restrictions on promotion of hand hygiene in the comparison group. Studies were excluded if they were not reported in peer reviewed publications or not written in English.

We applied a methodological filter by excluding studies that failed meet minimal quality criteria specified by the Cochrane Effectiveness Practice and Organisation of Care Group (EPOC). Acceptable study designs were randomised controlled trials and non-randomised trials (with at least two intervention and two control sites); controlled before-after studies (with outcome measures before and after the intervention from at least two intervention and two comparable control sites); and interrupted time series (with a clearly defined point in time for the intervention and outcome measures from at least three time points in both baseline and intervention periods).^12^ ^13^

### Patient involvement

No patients were involved in setting the research question or the outcome measures, nor were they involved in the design and implementation of the study. There are no plans to involve patients in dissemination.

### Data extraction and assessment of quality 

Two reviewers (NL and BSC) independently screened the titles and abstracts of the citations obtained from the search to assess the eligibility. Consensus was reached by discussion if initial assessments differed. NL evaluated the full text and abstracted data, which was checked by BSC.

The reviewers abstracted data including study design and duration, population, activities to promote hand hygiene in both intervention and comparison groups, hand hygiene outcomes, clinical and microbiological outcomes, measurement methods, and settings. When possible, we classified hand hygiene promotion activities according to WHO guidelines on hand hygiene in healthcare.^4^ We grouped activities into eight components: system change, education, feedback, reminders, safety climate, incentives, goal setting, and accountability (table 1). Results and raw compliance data from each study were extracted for further re-analyses. In addition, we extracted the costs of hand hygiene interventions or data on use of resources (materials and time spent on interventions) when appropriate. Additional information was obtained from the authors if it was not clear from the manuscript. For all included studies we used prespecified definitions to record the level of information (high, moderate, or low) about resources used for promotion of hand hygiene (see appendix 2).

**Table 1 tbl1:** Description of eight components of interventions to promote hand hygiene in healthcare workers

Component	Description
System change*	Ensuring necessary infrastructure is available including access to water, soap and towels and alcohol based handrub at point of care
Education and training	Providing training or educational programme on importance of hand hygiene and correct procedures for hand hygiene for healthcare workers
Feedback	Monitoring hand hygiene practices among healthcare workers while providing compliance feedback to staff
Reminders at workplace	Prompting healthcare workers either through printed material, verbal reminders, electronic communications or other methods, to remind them about importance of hand hygiene and appropriate indications and procedures
Institutional safety climate	Active participation at institutional level, creating environment allowing prioritisation of hand hygiene
Goal setting	Setting of specific goals aimed at improving compliance with hand hygiene, which can both apply at individual and group level and can include healthcare associated infection rates
Reward incentives	Interventions providing any reward incentive for participants completing a particular task or reaching a certain level of compliance. Both non-financial and financial rewards are included
Accountability	Interventions involved with improving healthcare workers’ accountability both at individual and unit level

*If the intervention period included changing the location or formulation of alcohol based handrub or installing more handrub dispensers, the baseline intervention was counted as no intervention or standard practice (no system change component), even if alcohol based handrub had been used during the baseline period.

### Assessment of risk of bias in included studies

We used the Cochrane Collaboration’s tool to assess risk of bias.^14^ Nine standard criteria for randomised controlled trials, non-randomised trials, and controlled before-after studies and seven standard criteria for interrupted time series were applied and used to classify each study’s risk of bias as low, high, or unclear.

### Data synthesis and statistical analysis

Data synthesis was performed separately for different study designs. The primary evidence synthesis was based on studies that used direct observation to measure compliance with hand hygiene. We restricted our analysis to this outcome because it reflects the opportunities for hand hygiene.

For randomised controlled trials, we used Cochrane Review Manager (RevMan; version 5.1) to calculate the natural logarithm of the odds ratio and associated variance to estimate the pooled odds ratio with a random effects model.^15^ The same method was applied to non-randomised trials, and controlled before-after studies if applicable. Heterogeneity between studies was assessed with the I^2 ^statistic. Risk of publication bias was evaluated with an enhanced contour funnel plot.^16^ ^17^

For interrupted time series, if re-analysis was required, we used a generalised linear segmented regression analysis to estimate the stepwise change in level and change in trend associated with the intervention.^18^ This approach is similar to that proposed by Ramsey and colleagues^19^ and Vidanapathirana and colleagues,^20^ except that it accounts for the binomial nature of the data, appropriately weighting each data point by the number of observations. We accounted for any evidence of autocorrelation by using Newey-West standard errors.^21^ Analysis was performed with Stata 13 (Statacorp LP, College Station, TX). We then estimated two summary measures that combined both stepwise and trend changes. Firstly, we calculated the mean natural logarithm of the odds ratio for hand hygiene associated with the intervention, a measure of relative improvement. Secondly, we calculated the mean percentage change in compliance in the period after the intervention (compared with that expected if there had been no intervention), an absolute measure of improvement in compliance. Standard errors were derived with the delta method by using the emdbook package in R.^22^ ^23^ Appendix 3 provides full details.

### Network meta-analysis

Network meta-analysis aims to combine all of the evidence, both direct and indirect, to estimate the comparative efficacy of all the interventions.^24^ Each intervention strategy is represented by a node in the network. If a study directly compares two interventions they are directly connected by a link on the network and a direct comparison is possible. If two interventions are connected indirectly (for example, if there are studies comparing each with a third intervention), then indirect comparison is possible.

We used network meta-analysis to compare the relative effectiveness of four different strategies: no promotion of hand hygiene, single component interventions, WHO-5, and WHO-5 and others (table 2). We included in the network meta-analysis those studies that included only these strategies and permitted a segmented regression analysis and directly observed compliance with hand hygiene.^25^ ^26^

**Table 2 tbl2:** Mean odds ratios with 95% credible intervals for interventions strategies to promote hand hygiene. Results are from random effects network meta-analysis model

Strategies*	Description	Mean OR (95% credible interval)
None/current practice	No intervention or current practice	Reference
Single intervention	Single intervention (system change or education)	4.30 (0.43 to 46.57)
WHO-5†	WHO-5 components	6.51 (1.58 to 31.91)
WHO-5* + others	WHO-5 plus incentives, goal setting, or accountability	11.83 (2.67 to 53.79)

*Model fit statistic: posterior mean residual deviance=10.40 and deviance information criterion (DIC)=23.86.

†Contained five components: system change, education, feedback, reminders, and institutional safety climate (see table 1 for details).

The effect sizes obtained from each comparison were combined in a network meta-analysis with a random effects model.^25^ Effect sizes were taken as the mean of the natural logarithm of the odds ratio for the hand hygiene intervention as estimated with the segmented regression model. Intervention rankings and associated credible intervals were obtained. Model fitting for the meta-analysis was carried out within a Bayesian framework using WinBUGS.^26^ Inconsistency checks were performed for closed loops in the network.^27^ Full model details are provided in appendix 4.

We performed a sensitivity analysis by excluding studies that implemented multicomponent strategies in a stepwise manner without sufficient data to evaluate individual components. This led to the exclusion of three studies.^28^ ^29^ ^30^

## Results

### Overall description

Figure 1 shows a summary of the review process[Fig fig1]. Of 3639 studies screened, 142 studies met initial inclusion criteria and 41 of these met EPOC criteria. Among these 41 studies, six were randomised controlled trials (including three cluster randomised controlled trials),^31^ ^32^ ^33^ ^34^ ^35^ ^36^ 32 were interrupted time series,^28^ ^29^ ^30^ ^37^ ^38^ ^39^ ^40^ ^41^ ^42^ ^43^ ^44^ ^45^ ^46^ ^47^ ^48^ ^49^ ^50^ ^51^ ^52^ ^53^ ^54^ ^55^ ^56^ ^57^ ^58^ ^59^ ^60^ ^61^ ^62^ ^63^ ^64^ ^65^ one was a non-randomised trial,^66^ and two were controlled before-after studies.^67^ ^68^ Appendix 5 give details of the reasons for exclusion. Applying our search strategy to three years covered by previous reviews did not yield any studies meeting our inclusion criteria that had not already been included.

**Figure fig1:**
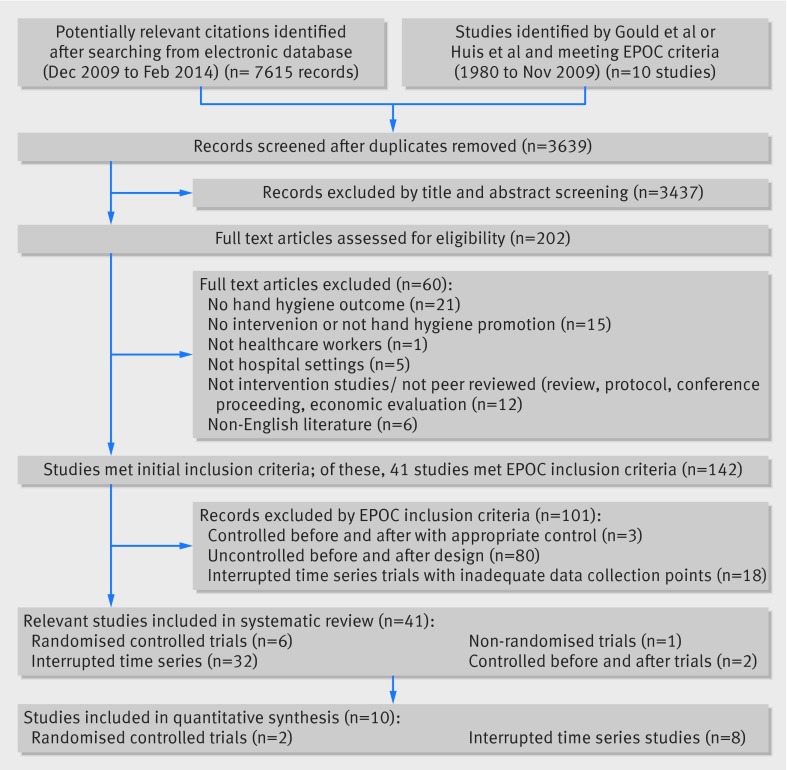
**Fig 1** Flow chart of study identification in systematic review of interventions to promote hand hygiene in healthcare workers

Seventeen studies applied interventions to the whole hospital, while 21 studies enrolled hospital wards. Three studies allocated interventions to specific healthcare workers.^31^ ^33^ ^36^ Twenty five studies were conducted in either a hospital-wide setting or combined intensive care units and general wards, while 11 were conducted in intensive care units or general wards alone. Of 10 studies conducted in more than one hospital, three included two or more countries.^42^ ^48^ ^50^ Only five of the 41 studies were conducted in low or middle income countries.^33^ ^36^ ^46^ ^50^ ^51^

Study periods ranged from two months to six years. In 11 studies the period was up to one year; in 17 studies it was more than a year and up to three years; and in 13 it was more than three years. Among the 32 interrupted time series, only 11 were longer than 12 months.

In 34 studies hand hygiene was observed in all types of healthcare workers with patient contact, while six studies considered only nurses and/or nursing assistants.^33^ ^34^ ^36^ ^60^ ^64^ ^68^ One study recruited only nursing students as participants.^54^ One study also included patients’ relatives.^39^

Six studies used a single faceted intervention: four implemented education alone^33^ ^46^ ^54^ ^68^ and two applied system change or reminders.^39^ ^44^ Seventeen studies used interventions equivalent to WHO-5, and six of these added supplemental interventions including goal setting, incentives, and accountability.^28^ ^34^ ^40^ ^45^ ^56^ ^66^ Nineteen studies implemented interventions with two to four components; four of these applied components not in WHO-5, including goal setting and incentives.^37^ ^38^ ^41^ ^59^

Thirty studies (four randomised controlled trials, 25 interrupted time series, and one non-randomised trial) used direct observation to measure compliance with hand hygiene. Two of these used a combination of video recorders and external observers.^37^ ^38^ Proxy measures were assessed in 19 studies including the rate of hand hygiene events, consumption of hand hygiene products (alcohol hand rub or soap), and a hand hygiene score checklist (two randomised controlled trials, 15 interrupted time series, and two controlled before and after studies). Clinical outcomes were reported in 19 studies.^28^ ^29^ ^30^ ^35^ ^42^ ^46^ ^47^ ^48^ ^49^ ^52^ ^55^ ^56^ ^57^ ^59^ ^62^ ^63^ ^66^ ^67^ ^69^ Appendix 6 provides full study characteristics including study design, setting, intervention, and comparison groups.

Examination of funnel plots (appendix 7) did not provide any clear evidence of publication bias, though evidence for or against such bias was limited by the fact that there were no more than four studies for any pairwise comparison of strategies.

### Quality assessment

Ten studies were considered to have a high risk of bias. Thirty one had either low or unclear risk. High risk of bias was present in all three non-randomised trials or controlled before-after studies but only in seven out of 32 interrupted time series. No randomised controlled trials or cluster randomised controlled trials were thought to have a high risk of bias (fig 2).

**Figure fig2:**
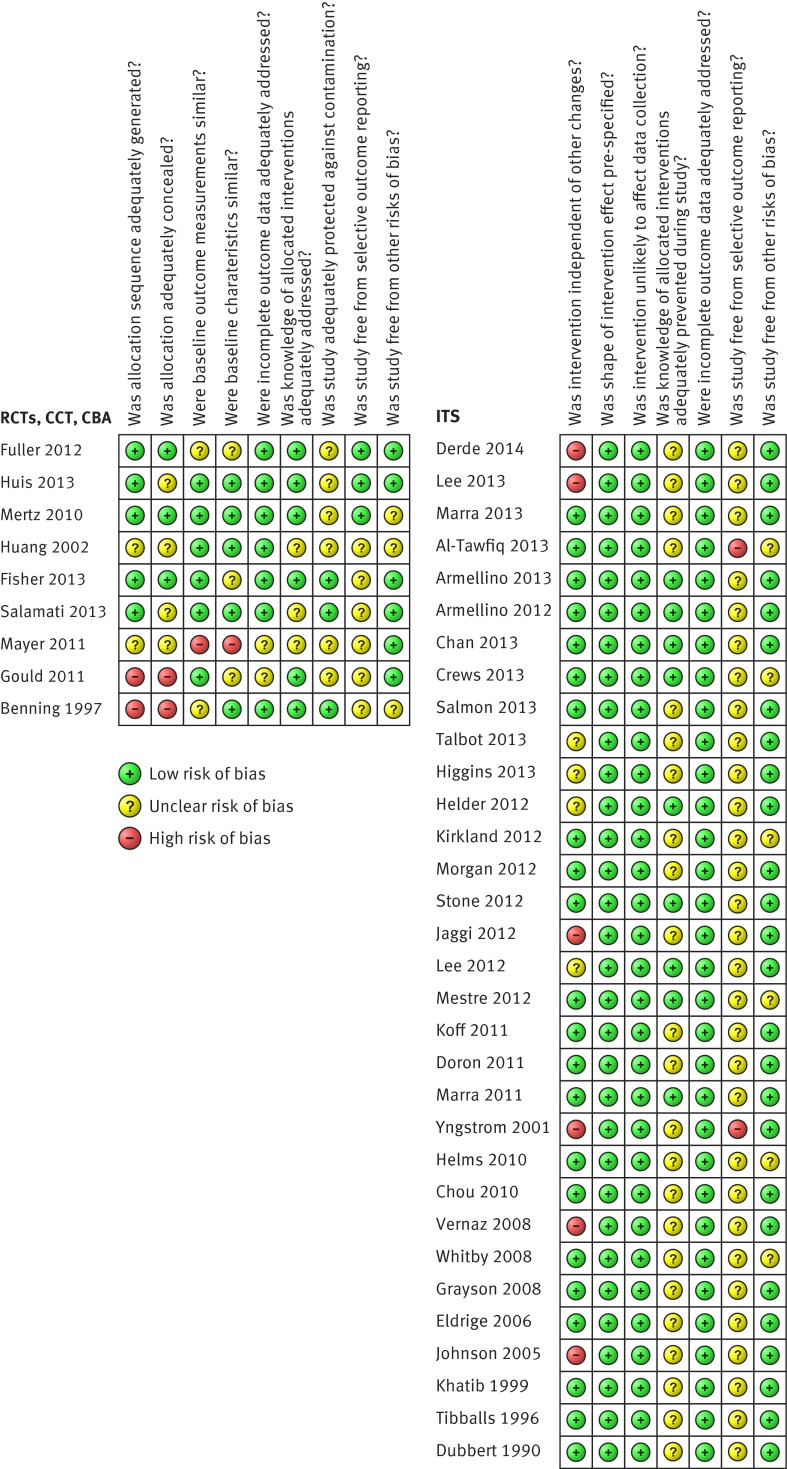
**Fig 2 **Assessment of risk of bias in included studies of interventions to promote hand hygiene in healthcare workers

The two controlled before-after studies^67^ ^68^ had high risks for inadequate allocation sequence and concealment, while one non-randomised trial^66^ had high risk of dissimilarity in baseline outcome between experimental and control groups.

Fourteen studies (34%) had a low risk of bias due to the knowledge of allocated intervention, as these studies either measured objective outcomes (such as alcohol consumption or output from electronic counting devices) or stated that the observers were blinded to the intervention. The rest of the studies had unclear risk as they did not report whether the observers were blinded.

Risk of selective outcome reporting was unclear in 33 studies as pre-specified protocols were reported only in three randomised controlled trials.^32^ ^34^ ^35^ Two of the interrupted time series had a high risk of selective outcome reporting as they reported on a non-periodical basis.^28^ ^59^ Among the interrupted time series, six had a high risk that outcomes were affected by other interventions such as a universal chlorhexidine body washing programme,^42^ ^63^ reinforcement of standard precautions,^42^ screening and decolonisation for multidrug-resistant micro-organisms,^48^ quality improvement program,^46^ ^59^ and antibiotic use and healthcare associated infections control policy implemented at the same time.^56^

### Meta-analysis/data synthesis

#### Randomised controlled trials

Four of six randomised controlled trials measured compliance with hand hygiene by direct observation with indications similar to WHO-5.^32^ ^33^ ^34^ ^35^ Two of these studies compared WHO-5 with WHO-5 combined with goal setting (WHO-5+).^32^ ^34^ Huis and colleagues performed a cluster randomised trial in 67 wards from three hospitals in the Netherlands.^34^ Compliance immediately after the intervention increased from 23% to 42% in the WHO-5 arm and from 20% to 53% in the WHO-5+ arm; in both arms improvements were sustained six months later. Fuller and colleagues used a three year stepped wedge design in 16 intensive care units and 44 acute care of the elderly wards and reported an absolute increase in compliance of 13-18% and 10-13%, respectively, in implementing wards.^32^ Only 33 of 60 enrolled wards, however, implemented the intervention (22 out of 44 elderly wards and 11 out of 16 intensive care units), and the intention to treat analysis did not show increased compliance in the elderly wards while compliance in intensive care units increased by 7-9%. Meta-analysis (with intention to treat results) provided evidence favouring the WHO-5+ strategy. The pooled odds ratio was 1.35 (95% confidence interval 1.04 to 1.76; I^2^=81%) (fig 3). The large heterogeneity seemed to be caused by the low fidelity to intervention in acute care of the elderly wards. Per protocol analyses gave similar odds ratios for compliance to the study by Huis and colleagues (1.67 (95% confidence interval 1.28 to 2.22) for elderly wards and 2.09 (1.55 to 2.81) for intensive care units). Two other randomised controlled trials directly reported observed compliance with hand hygiene. An individually randomised trial of an education programme versus no intervention for nurses in China reported an absolute improvement in compliance of 32.7% (95% confidence interval 15.6% to 49.7%) for opportunities before contact with patients and 20.4% (5.6% to 35.2%) for opportunities after contact (baseline compliance before and after contact was about 25% and 37%, respectively, in both arms).^33^ In Canada, a cluster randomised trial of a bundle of education, performance feedback, and visual reminders in 30 hospital units where alcohol hand rub was available at point of care in both arms (but with no other interventions in the control arm) reported a higher adherence after the intervention in the intervention arm (mean difference 6.3%, 95% confidence interval 4.3% to 8.4%).^35^ In both arms baseline compliance was low (16%).

**Figure fig3:**
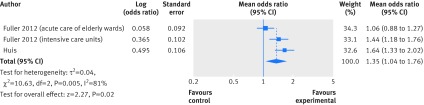
**Fig 3** Forest plot of the associations between WHO-5 and goal setting compared with WHO-5 alone and compliance with hand hygiene from randomised controlled trials using intention to treat results

Fisher and colleagues randomised individuals to either a control group where hand hygiene was not actively promoted or an intervention arm that used audio reminders and individual feedback.^31^ They assessed compliance using an automated system at entry to and exit from patients’ rooms. The intervention was associated with a 6.8% (95% confidence interval 2.5% to 11.1%) improvement in compliance. Salamati and colleagues randomised nursing personnel to either a motivational interviewing intervention (a behaviour modification approach initially developed to treat patients with alcoholism) or a control group.^36^ Both arms also received an educational intervention. The outcome measure was a composite hand hygiene score, which was found to increase in the intervention arm. The scoring details, however, were unclear.

### Interrupted time series

Of 32 interrupted time series, 25 measured hand hygiene compliance. Only 18 studies with direct observation, however, reported the number of observations at each time point, making them eligible for re-analysis.^28^ ^29^ ^30^ ^37^ ^38^ ^40^ ^41^ ^42^ ^43^ ^45^ ^46^ ^48^ ^50^ ^54^ ^56^ ^60^ ^64^ ^65^ As some of these studies were conducted at multiple sites^48^ or had multiple intervention phases,^56^ 22 pairwise comparisons from these 18 studies were available for re-analysis (fig 4). In four studies there was evidence of positive first order autocorrelation.^37^ ^38^ ^40^ ^56^

**Figure fig4:**
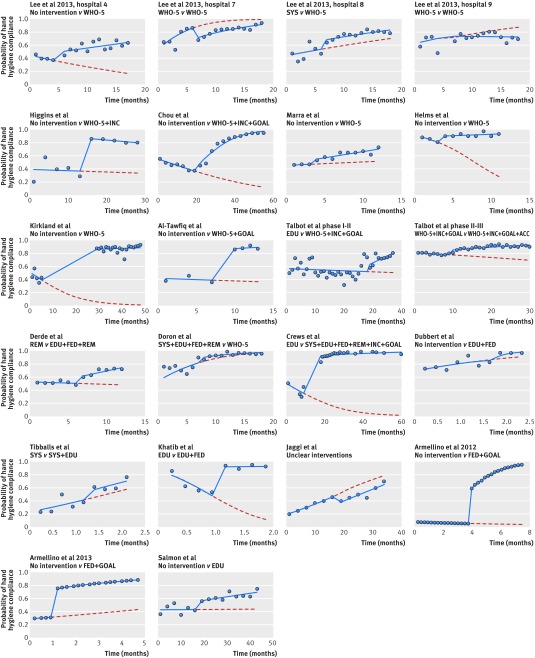
**Fig 4** Re-analysis of studies involving interrupted time series where the outcome was hand hygiene compliance. Points represent observations, solid lines show expected values from fitted segmented regression models, and broken lines represent extrapolated trends before intervention. SYS=system change; EDU=education; FED=feedback; REM=reminders; SAF=institutional safety climate; INC=incentives; GOAL=goal setting; ACC=accountability; WHO-5=combined intervention strategies including SYS, EDU, FED, REM, and SAF

The baseline compliance ranged from 7.6% to 91.3%. Twelve of 22 comparisons showed a declining trend in compliance during the period before intervention; seven of these did not report any activities to promote hand hygiene before intervention, while another four used only education or reminders. Fifteen pairwise contrasts showed a positive change in trend for compliance with hand hygiene after the intervention (table 3). All but four contrasts showed both stepwise increases in compliance with hand hygiene associated with the intervention and increases in mean compliance in the period after intervention compared with that expected in the absence of the intervention. The range was wide: the mean change in hand hygiene attributed to the intervention varied between a decrease of 14.8% and an increase of 83.3% (table 3). Two studies had an intervention period lasting at least two years; neither showed evidence for any decline in compliance over this period.^40^ ^41^ In only one study was there a net trend for decreasing compliance after the intervention (fig 4).^45^

**Table 3 tbl3:** Results of re-analysis of studies using interrupted time series to assess compliance with hand hygiene

Study	Comparison	Baseline (intercept)		Coefficient (SE) for baseline trend	Coefficient (SE) for change in trend	Coefficient (SE) for change in level	Mean (95% CI)* % change in compliance
		% compliance	Coefficient (SE)				
Lee^48^							
Hospital 4	No intervention *v* WHO-5	44.6	−0.215 (0.30)	−0.081 (0.10)	0.130 (0.10)	0.606 (0.26)	29.9 (3.5 to 56.4)
Hospital 7	WHO-5 *v* WHO-5	53.8	0.154 (0.29)	0.281 (0.07)	−0.151 (0.08)	−1.042 (0.25)	−11.5 (−13.5 to −9.5)
Hospital 8	SYS *v* WHO-5	44.6	−0.215 (0.26)	0.059 (0.06)	0.014 (0.06)	0.563 (0.19)	13.3 (−9.2 to 35.8)
Hospital 9	WHO-5 *v* WHO-5	62.3	0.503 (0.33)	0.088 (0.13)	−0.094 (0.13)	−0.007 (0.51)	−9.7 (−63.6 to 44.3)
Derde^42^	REM *v* EDU+FED+REM	52.8	0.112 (0.04)	−0.015 (0.01)	0.133 (0.02)	0.346 (0.05)	16.3 (13.6 to 19.1)
Higgins^45^	No intervention *v* WHO-5+INC	37.2	−0.428 (0.17)	−0.009 (0.25)	−0.030 (0.03)	2.448 (0.25)	48.8 (45.4 to 52.3)
Doron^43^	SYS+EDU+FED+REM *v* WHO-5	70.7	0.204 (0.12)	0.187 (0.10)	−0.040 (0.03)	0.586 (0.01)	4.7 (2.3 to 7.1)
Chou^40^†	No intervention *v* WHO-5+INC+GOAL	54.9	0.198 (0.03)	−0.039 (0.00)	0.151 (0.01)	0.453 (0.17)	56.4 (53.1 to 59.8)
Marra^50^	No intervention *v* WHO-5	45.7	−0.173 (0.07)	0.020 (0.06)	0.063 (0.03)	0.218 (0.06)	11.5 (3.4 to 19.6)
Helms^30^	No intervention *v* WHO-5	91.3	2.350 (0.42)	−0.297 (0.18)	0.354 (0.19)	0.706 (0.33)	35.9 (−5.8 to 77.7)
Kirkland^29^	No intervention *v* WHO-5	51.3	0.052 (0.14)	−0.097 (0.04)	0.111 (0.04)	4.443 (1.03)	83.3 (77.0 to 89.6)
Al-Tawfiq^28^	No intervention *v* WHO-5+GOAL	41.3	−0.350 (0.09)	−0.014 (0.02)	0.081 (0.07)	2.328 (0.21)	49.9 (42.8 to 57.0)
Crews^41^	EDU *v* SYS+EDU+FED+REM+INC+GOAL	50.7	0.028 (0.12)	−0.070 (0.02)	0.103 (0.02)	3.679 (0.22)	38.2 (35.5 to 40.9)
Talbot (phase I)^56^†	EDU *v* WHO-5+INC+GOAL	56.7	0.271 (0.20)	−0.006 (0.02)	0.109 (0.02)	0.363 (0.41)	18.5 (−1.4 to 38.4)
Talbot (phase II)^56^	WHO-5+INC+GOAL *v* WHO-5+INC+GOAL+ACC	81.1	1.455 (0.45)	−0.020 (0.01)	0.060 (0.01)	0.464 (0.05)	15.0 (10.6 to 19.5)
Dubbert^60^	No intervention *v* EDU+FED	69.5	0.822 (0.34)	0.636 (0.39)	2.908 (1.57)	−0.753 (0.75)	0.7 (−10.0 to 11.4)
Tibballs^65^	SYS *v* SYS+EDU	23.4	−1.186 (0.53)	0.187 (0.10)	−0.040 (0.03)	0.453 (0.57)	11.9 (−18.4 to 42.1)
Khatib^64^	EDU *v* EDU+FED	86.2	1.836 (0.17)	−2.051 (0.26)	2.185 (0.52)	2.549 (0.29)	65.8 (58.6 to 73.0)
Jaggi^46^	Unclear intervention details	19.5	−1.420 (0.26)	0.080 (0.02)	−0.006 (0.03)	−0.586 (0.34)	−14.8 (−33.1 to 3.6)
Armellino^38^†	No intervention *v* FED+GOAL	7.6	−2.493 (0.15)	−0.088 (0.133)	0.849 (0.235)	3.046 (0.68)	45.4 (38.5 to 52.3)
Armellino^37^†	No intervention *v* FED+GOAL	29.0	−0.895 (0.04)	0.122 (0.10)	−0.109 (0.08)	2.267 (0.14)	74.9 (65.5 to 84.4)
Salmon^54^‡	No intervention *v* EDU	42.7	−0.295 (0.17)	0.003 (0.02)	0.021 (0.02)	0.485 (0.22)	17.9 (−0.3 to 36.2)

SYS=system change; EDU=education; FED=feedback; REM=reminders; SAF=institutional safety climate; INC=incentives; GOAL=goal setting; ACC=accountability; WHO-5=combined intervention strategies including SYS, EDU, FED, REM, and SAF.

*Mean change in hand hygiene compliance during period after intervention period attributed to intervention accounting for baseline trends (see appendix 3 for details)

†Evidence of autocorrelation; Newey-West standard errors reported.

‡Hand hygiene compliance measured in student nurses.

### Non-randomised trials and controlled before-after studies

Mayer and colleagues compared WHO-5 and reward incentives (WHO-5+) with a combination of system change, education, and feedback using a staggered introduction of an intervention bundle, across four out of six patient units.^66^ The WHO-5+ intervention was associated with improved compliance, which increased from 40% to 64% in one two-unit cohort and from 34% to 49% in the other. 

Benning and colleagues reported a hospital-wide trend of increased soap and alcohol consumption in both intervention (package of system change, reminders, and safety climate) and control (no intervention) groups but found no evidence of an increased effect in the intervention group.^67^ Gould and colleagues found no evidence of improvement in frequency of hand decontamination in surgical intensive care wards resulting from a series of educational lectures compared with no intervention (control).^68^

### Analysis of interrupted time series and network meta-analysis

Among the 22 pairwise comparisons from interrupted time series, 18 had clear details about interventions and similar indications for compliance with hand hygiene among qualified healthcare workers. In 16 of these the intervention period included additional intervention components alongside measures to promote hand hygiene used in the baseline period, and all outcome data favoured the intervention (fig 5). In the two comparisons where there was no improvement in hand hygiene, all components of the intervention were already in place in the baseline period.^48^

**Figure fig5:**
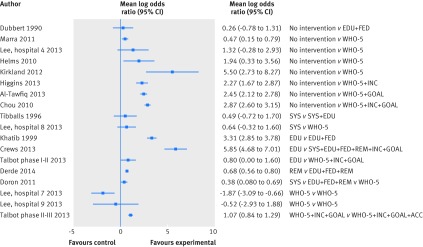
**Fig 5** Forest plot showing effect size as mean log odds ratios for hand hygiene compliance for all direct pairwise comparisons from interrupted time series studies. Lee and colleagues^48^ was a multi-centre study. In hospitals 8 and 9 baseline strategy was already equivalent to WHO-5. SYS=system change; EDU=education; FED=feedback; REM=reminders; SAF=institutional safety climate; INC=incentives; GOAL=goal setting; ACC=accountability; WHO-5=combined intervention strategies including SYS, EDU, FED, REM, and SAF

Twelve pairwise comparisons met the criteria for network meta-analysis, and included direct comparisons between all pairs of strategies except WHO-5 versus WHO-5+ and no intervention versus single intervention (fig 6). The network meta-analysis showed that although there was large uncertainty in effect sizes among the pairwise comparisons, point estimates for all intervention strategies indicated an improvement in compliance with hand hygiene compared with no intervention (fig 7). When two strategies, WHO-5 and WHO-5+, were compared with no intervention there was strong evidence that they were effective (table 2). The WHO5+ strategy also showed additional improvement compared with single intervention strategies and WHO-5 alone. For the latter comparison, which depended only on indirect comparisons, the estimated effect size was similar to that seen in the randomised controlled trials, though uncertainty was much larger (odds ratio for WHO-5 versus WHO-5+ was 1.82, 95% credible interval 0.2 to 12.2). WHO-5+ had the highest probability (67%) of being the best strategy in improving compliance (fig 8).

**Figure fig6:**
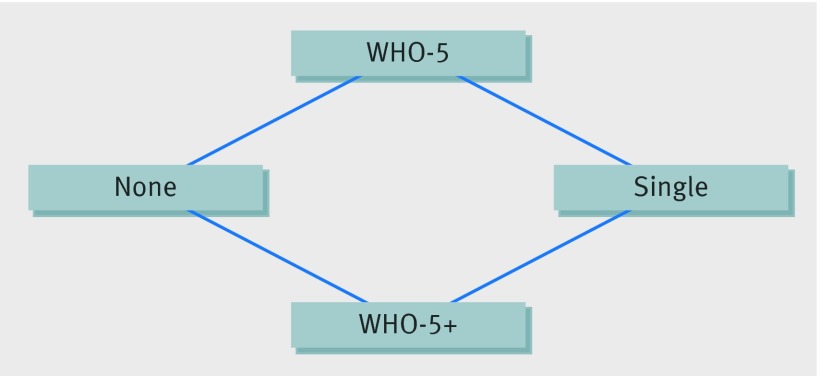
**Fig 6** Network structure for network meta-analysis of four hand hygiene intervention strategies from interrupted time series studies. Intervention strategies were: none (no intervention); single intervention; WHO-5; and WHO-5+ (WHO-5 with incentives, goal-setting, or accountability)

**Figure fig7:**
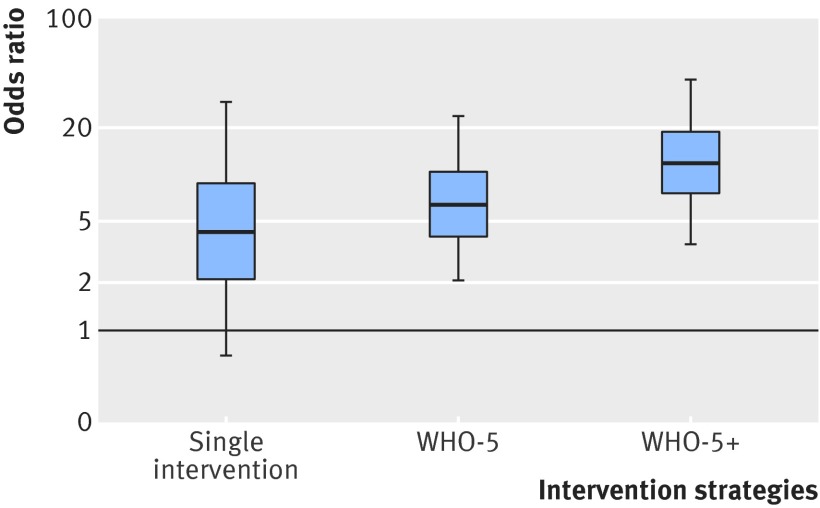
**Fig 7** Box-and-whiskers plot showing relative efficacy of different hand hygiene intervention strategies compared with standard of care estimated by network meta-analysis from interrupted time series studies. Lower and upper edges represent 25th and 75th centiles from posterior distribution; central line median. Whiskers extend to 5th and 95th centiles. Intervention strategies were single intervention; WHO-5; and WHO-5+ (WHO-5 with incentives, goal-setting, or accountability). Appendix 9 shows results from sensitivity analysis that excluded studies where interventions were implemented as multiple time points

**Figure fig8:**
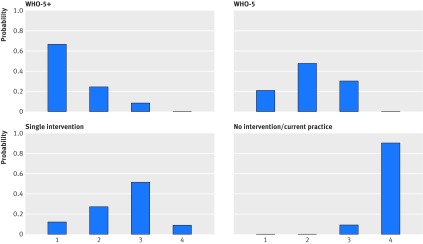
**Fig 8** Rankograms showing probabilities of possible rankings for each intervention strategy (rank 1=best, rank 4=worst)

After we excluded studies with multiple stepwise interventions in the sensitivity analysis, there was a decrease in the effect size of all intervention strategies (appendix 4).

### Clinical outcomes

Nineteen studies reported clinical or microbiological outcomes alongside hand hygiene outcomes. Six of these were multicentre studies,^35^ ^42^ ^48^ ^55^ ^62^ ^67^ and 13 were based in a single hospital.^28^ ^29^ ^30^ ^46^ ^47^ ^49^ ^52^ ^56^ ^57^ ^59^ ^63^ ^66^ ^69^ All reported that improvements in hand hygiene were associated with reductions in at least one measure of hospital acquired infection and/or resistance rates. In most case, however, either appropriate analysis was lacking, denominators were not reported, time series data were not shown (making interrupted time series designs vulnerable to pre-existing trends), or numbers were too small to draw firm conclusions.

There were, however, three single centre studies that did not have these limitations.^49^ ^57^ ^63^ Two of these studies, which lasted about seven years, used time series analysis to study associations between use of alcohol hand rub and clinical outcomes, with adjustment for changing patterns of antibiotic use.^49^ ^57^ Lee and colleagues found strong evidence (P<0.001) that increased use of alcohol hand rub was associated with reduced incidence of healthcare associated infection and evidence that it was associated with reduced healthcare associated methicillin resistant *Staphylococcus aureus* (MRSA) infection (P=0.02).^49^ Vernaz and colleagues found strong evidence that increased use of alcohol based hand rub was associated with reduced incidence of MRSA clinical isolates per 100 patient days (P<0.001), reporting that 1L of hand rub per 100 patient days was associated with a reduction in MRSA of 0.03 isolates per 100 patient days.^57^ No association was found between increased use of alcohol based hand rub and clinical isolates of *Clostridium difficile*. Johnson and colleagues reported that an intervention in an Australian teaching hospital associated with a mean improvement of compliance with hand hygiene from 21% to 42% was also associated with declining trends in clinical MRSA isolates (by 36 months after the intervention clinical isolates per discharge had fallen by 40% compared with the baseline before the intervention), declining trends in MRSA bacteraemias (57% lower than baseline after 36 months), and declining trends in clinical isolates of extended spectrum β lactamases (ESBL) producing *E coli* and *Klebsiella* (>90% below baseline 36 months after intervention), though there was no evidence of changes in patient MRSA colonisation at four or 12 months after the intervention.^63^ In addition to hand hygiene, however, the intervention included patient decolonisation and ward cleaning, and the relative importance of these measures cannot be determined.

Among the multicentre studies, Grayson and colleagues described a similar hand hygiene intervention (but without additional decolonisation or ward cleaning) initially introduced to six hospitals as a pilot study and, later, to 75 hospitals in Victoria, Australia, as part of a state-wide roll out.^62^ Both the pilot and roll out were associated with large improvements in compliance (from about 20% to 50%) and similar clinically important trends after the intervention for reduced MRSA bacteraemias and MRSA clinical isolates per patient discharge (though in the state-wide roll out hospitals there was also a decline in MRSA clinical isolates before the intervention that continued after the intervention).

Roll out of a similar hand hygiene intervention (the Cleanyouhands campaign, based on WHO-5) in England and Wales was reported to be associated with reduced rates of MRSA bacteraemia (from 1.9 to 0.9 cases per 10 000 bed days) and *C difficile* infection (from 16.8 to 9.5 cases per 10 000 bed days), but no association was found with methicillin-sensitive *S aureus* (MSSA) bacteraemia.^55^ This study also reported independent associations between procurement of alcohol hand rub and MRSA bacteraemias; in the last 12 months of the study, MRSA bacteraemias were estimated to have fallen by 1% (95% confidence interval 5% to 15%) for each additional mL of hand rub used per bed day (adjusted for other interventions and hospital level mupirocin use, a surrogate marker for MRSA screening and decolonisation). Similarly, each additional mL of soap used per bed day was associated with a 0.7% (0.4%, 1.0%) reduction in *C difficile* infection.

Benning and colleagues described the evaluation of a separate but contemporaneous patient safety intervention that included a hand hygiene component in nine English hospitals with nine matched controls.^67^ Both intervention and control sites experienced large increases in consumption of soap and alcohol hand rub between 2004 and 2008 and substantial falls in rates of MRSA and *C difficile* infection, though in all cases (soap, hand rub, and infections) there was no evidence that differences between intervention and control sites resulted from anything other than chance.

In a two year study in 33 surgical wards in 10 European hospitals, Lee and colleagues found that, after adjustment for clustering, potential confounders, and temporal trends, enhanced hand hygiene alone was not associated with a reduction in MRSA clinical cultures and MRSA surgical site infections, and neither was a strategy of screening and decolonisation, but in wards where both interventions were combined, there was a reduction in the rate of MRSA clinical cultures of 12% per month (adjusted incidence rate ratio 0.88, 95% confidence interval 0.79 to 0.98).^48^

Among the randomised controlled trials, Mertz and colleagues found similar rates of hospital acquired MRSA colonisation in intervention and control groups (0.73 *v* 0.66 events per 1000 patient days, respectively; P=0.92), though adherence to hand hygiene was only 6% higher in the intervention arm.^35^ Finally, in a study in 13 European intensive care units, Derde and colleagues reported a declining trend in acquisition of antimicrobial resistant bacteria (weekly incidence rate ratio 0.976, 95% confidence interval 0.954 to 0.999) associated with a hand hygiene intervention that increased compliance from about 50% to over 70%.^42^ The decline was largely because of reduced MRSA acquisition. The intervention also included universal chlorhexidine body washing, and it is not possible to establish the relative importance of hand hygiene.

### Level of information on resource use

Reporting of information on cost and resource use was limited, with 3, 26, and 12 studies classified as having high, moderate, and low information, respectively (appendix 8). Three studies reported costs associated with both materials and person time^34^ ^52^ ^66^; in two cases these reports were in separate papers.^70^ ^71^ Table 4 summarises the reported costs of interventions[Table tbl4].

**Table 4 tbl4:** Extracted data on resource use in studies of interventions to improve hand hygiene in healthcare workers

Author (year), design	Intervention	Comparison	Settings and base year	Resource use		Sources	Total cost ($)*	No of beds	Intervention period (day)	Cost per 1000 bed day ($)
				Materials	Time					
Huis (2013), CRCT	WHO-5 + goal-setting	WHO5 (except institutional safety climate)	Netherlands, 2009	State of art strategies: alcohol hand rub, website, leaflets, posters, newsletters, article in hospital magazines	State of art strategies: hand hygiene, direct observation. Extra time for performing hand hygiene	Separate paper^70^	320 278	993	365	883.7
				Team and leader directed strategies: as above	Team and leader directed strategies: as above plus coach salary, staffing costs for managers, role models and nurses in coaching session and preparation		474 068	1225	365	1060.5
Higgins (2013), ITS	WHO-5 + incentive	None (with AHR)	Ireland, 2010	Mobile interactive stand-alone computer using gaming technology and annual license. Swab and ATP machine	Research assistant for audit and training 1.79 FTE (287 hours) assuming salary as £2500 per month	Author contact	42 358	170	450	553.7
Armellino (2012), ITS	Feedback + goal-setting	None (unclear AHR use)	USA, 2008	21 video cameras	N/A	Paper	50 000	17	630	4668.5
Morgan (2012), ITS	System change + education + feedback + reminders	None (with AHR)	USA, 2010	60 alcohol dispensers system in two wards, 12 posters in total	1.46 FTE (234 hours) research assistants (10-20 hours/week for trouble shooting, refilling, and collecting data from devices, and 2 hours/month to design and present posters)	Author contact	6960	27	105	2455.0
Mestre (2012), ITS	Phase I: WHO-5. Phase II: WHO-5 (intense) + Reinforcement	Phase I: None (with AHR); phase II: WHO-5	Spain, 2011	Alcohol handrub solution. Material for campaign including posters, pens, and candy	Hand hygiene direct observation. Data analysis and interpretation	Separate paper^71^	19 259	n/a	365	385.2
Doron (2011), ITS	WHO-5	System change + education + feedback + reminders (with AHR)	USA, 2008-9	Cost for marketing consultancy	N/A	Author contact	35 000-50 000	425	365	225.6-322.3
Mayer (2011), NRT	Phase I: system change + education + feedback. Phase II: WHO-5 + incentive	None (unclear AHR use)	USA, 2003-6	Prizes as candy, chocolate bars, pizza, and others	2.25 FTE (yearly) of infection preventionists; 0.6 FTE of manager 0.35 FTE of clerk	Paper	165 600	450	365	1008.2

AHR=alcohol based hand rub, CRCT=cluster randomized controlled trial, ITS=interrupted time series, NRT= non-randomized trial, WHO-5=combined intervention including system change, education, feedback, reminders, and institution safety climate, N/A= not available, FTE=full time equivalents.

*$1=£0.65, €0.90.

## Discussion

### Principal findings

A multi-faceted hand hygiene intervention—WHO-5—and single interventions including system change, training and education, or reminders alone are associated with improved compliance with hand hygiene among healthcare workers in hospital compared with standard practice. Results from both randomised controlled trials and interrupted time series designs provide consistent evidence that adding supplemental interventions including goal setting, reward incentives, and accountability to the WHO-5 strategy lead to additional improvements in compliance. Information about resources used in the interventions was not well reported.

### Comparison with other studies

We are aware of four previous systematic reviews of interventions for hand hygiene in healthcare settings.^5^ ^6^ ^7^ ^8^ One of these found only four studies of sufficient methodological quality to reliably evaluate interventions to promote hand hygiene and was unable to reach firm conclusions.^5^ Overlap between included studies in the other three and our review is small: respectively four (9.8%),^8^ three (7.3%),^6^ and five (12.2%)^7^ of studies included in our review were included in previous reviews, while 17 (80.1%), 38 (92.7%), and 40 (88.9%) of the studies in these reviews failed to meet the minimum quality threshold in ours.^12^ ^13^ While high quality non-randomised studies can potentially play an important role in the evaluation of interventions if they are analysed with appropriate methods, there are many reasons for thinking that simple before-after studies (a design used by most of the studies included in previous reviews) do not provide a reliable basis for evaluating interventions.^72^ ^73^ ^74^ While an interrupted time series study (where multiple outcome measures are taken before and after the intervention) represents a strong quasi-experimental design, a before-after study compares a single outcome measure before and after the intervention and is vulnerable to distorting effects of pre-existing trends.

We found an increasing number of “high quality” studies on interventions for hand hygiene after 2009. From two previous systematic reviews^5^ ^6^ examining the literature from 1980 to November 2009, we found only 10 studies meeting the EPOC criteria (one randomised controlled trial, eight interrupted time series, and one controlled before-after study). With the same criteria, our review found 31 studies (five randomised controlled trials, 24 interrupted time series, one non-randomised trial, and one controlled before-after study) published between December 2009 and February 2014.

Reporting on resource implications for interventions was generally limited with some notable exceptions. Most included studies reported only part of the resources used, and methods for collecting cost data were unclear. Such information on resource use is important both for those wishing to implement similar strategies and for economic evaluation of different interventions.^10^ ^75^ A good framework to collect such data has also been proposed.^76^ Cost effectiveness analysis of promotion of hand hygiene is required to assess under what circumstances these initiatives represent good value for money and when resources might be better directed at supplemental interventions, including care bundles,^77^ ward cleaning,^78^ and screening and decolonisation,^79^ to complement well maintained compliance with hand hygiene.

### Strengths and limitations of study

A particular strength of our study is that the network meta-analysis allowed us to quantify the relative efficacy among a series of different intervention strategies with different baseline interventions, even where the direct head-to-head comparisons were absent.

This study also has several limitations. Firstly, details on implementation of components of the intervention varied substantially. For example, personal feedback and group feedback were classified together, but, in practice, the impacts of these strategies can vary. Moreover, different studies might implement the same programme with different quality of delivery and level of adherence, so called intervention fidelity or type III error.^80^ Both issues are common to many interventions to improve the quality of care in hospital settings and are likely to be responsible for much of the unexplained heterogeneity between studies.^81^ ^82^ Secondly, direct observation of compliance with hand hygiene might induce an increase in compliance unrelated to the intervention (the Hawthorne effect). Recent research suggests that such Hawthorne effects can lead to substantial overestimation of compliance.^83^ ^84^ Such effects, however, should not bias estimates of the relative efficacy of different interventions from randomised controlled trials and interrupted time series unless the effects vary between study arms/intervention periods. Thirdly, it is possible that it is the novelty of the intervention itself that leads to improvements in compliance and that any sufficiently novel intervention would do the same regardless of the components used. This clearly cannot be ruled out and is not necessarily inconsistent with our findings that interventions with more components tend to perform better. At present, however, there are too few high quality studies to evaluate whether individual components of interventions show consistent differences that cannot be explained by novelty alone. Fourth, results might be distorted by publication bias. Fifth, there might also be a low level of language bias because we excluded studies in languages other than English. The magnitude of such bias, however, is likely to be small.^85^ ^86^

Finally, linking improved compliance to clinical outcomes such as number of infections prevented would provide more direct evidence about the value of such interventions.^10^ Such direct evidence is still limited in hospital settings, although the association is supported by a growing body of indirect evidence as well as biological plausibility. Moreover, findings from studies included in our review that reported clinical or microbiological outcomes are consistent with substantial reductions in infections for some pathogens, such as MRSA, resulting from large improvements in hand hygiene.^87^ ^88^ The lack of a measureable effect of improved hand hygiene on MSSA infections might seem paradoxical but can be partly explained by the fact that MSSA infections are much more likely to be of endogenous origin, whereas MRSA is more often linked to nosocomial cross transmission. Moreover, predictions from modelling studies that hand hygiene will have a disproportionate effect on the prevalence of resistant bacteria in hospitals (provided resistance is rare in the community) seem to have been borne out in practice.^89^

### Conclusions

While there is some evidence that single component interventions lead to improvements in hand hygiene, there is strong evidence that the WHO-5 intervention can lead to substantial, rapid, and sustained improvements in compliance with hand hygiene among healthcare workers in hospital settings. There is also evidence that goal setting, reward incentives, and accountability provide additional improvements beyond those achieved by WHO-5. Important directions for future work are to improve reporting on resource implications for interventions, increasingly focus on strong study designs, and evaluate the long term sustainability and cost effectiveness of improvements in hand hygiene.

**What is already known on this topic**

Hand hygiene among healthcare workers is possibly one of the most effective measures to reduce healthcare associated infections, but compliance remains poor in many hospital settings
In 2005 WHO launched a campaign to improve hand hygiene in healthcare settings by promoting a multimodal strategy consisting of five components: system change, training and education, observation and feedback, reminders in the hospital, and a hospital safety climate

**What this study adds**

These meta-analyses provide evidence that the WHO campaign is effective at increasing compliance with hand hygiene in healthcare workers
There is evidence that additional interventions (used in conjunction with the WHO campaign elements), including goal setting, reward incentive, and accountability, can lead to further improvements
Reporting on resource implications of such interventions is limited

## Notes

We are grateful to all authors of the literature included in this review who responded to requests for additional information. We also thank the infection control staff of Sappasithiprasong Hospital for technical support and Cecelia Favede for help in editing.

Ethical approval: Not required

Data sharing: The relevant data and code used in this study are available from the authors.

Transparency: The lead author affirms that this manuscript is an honest, accurate, and transparent account of the study being reported; that no important aspects of the study have been omitted; and that any discrepancies from the study as planned (and, if relevant, registered) have been explained.

## Notes

Cite this as: *BMJ* 2015;351:h3728
