# Supplementary material for: Comparative efficacy of interventions to promote hand hygiene in hospital: systematic review and network meta-analysis
Source: BMJ. 2015 Jul 28;351:h3728. doi: 10.1136/bmj.h3728 (PMC4517539; doi:10.1136/bmj.h3728)
Supplement: Supplementary file 1 [file luan024450.ww1_default.pdf]

Appendix 1: Electronic search strategy [posted as supplied by author]

Databases	Adapted from Gould et al.	Adapted from Huis et al.
MEDLINE	1 Handwashing/<br>2 (hand antisepsis or handwash$ or hand wash$ or hand disinfection or hand hygiene or surgical scrub$).tw.<br>3 1 or 2<br>4 exp Hand/<br>5 exp Sterilization/<br>6 4 and 5<br>7 3 or 6<br>8 randomized controlled trial.pt.<br>9 controlled clinical trial.pt.<br>10 intervention studies/<br>11 experiment$.tw.<br>12 (time adj series).tw.<br>13 (pre test or pretest or (posttest or post test)).tw.<br>14 random allocation/<br>15 impact.tw.<br>16 intervention?.tw.<br>17 chang$.tw.<br>18 evaluation studies/<br>19 evaluat$.tw.<br>20 effect?.tw.<br>21 comparative study/<br>22 animal/<br>23 human/<br>24 22 not 23<br>25 or/8-21<br>26 25 not 24<br>27 7 and 26<br>28 limit 27 to yr="2009 -Current"<br>29 exp hospitals/<br>30 hospital$.tw.<br>31 exp inpatients/<br>32 inpatient$.tw.<br>33 exp health care/<br>34 health care$.tw.<br>35 healthcare$.tw.<br>36 infirmary$.tw.<br>37 nosocomial$.tw.<br>38 intensive care unit$.tw.<br>39 ward$.tw.<br>40 OR/29-39<br>41 28 and 40	1 Randomized controlled trial/<br>2 random$.tw.<br>3 experiment$.tw.<br>4 (time adj series).tw.<br>5 (pre test or pretest or post test or posttest).tw.<br>6 impact.tw.<br>7 intervention$.tw.<br>8 chang$.tw.<br>9 evaluat$.tw.<br>10 effect?.tw.<br>11 compar$.tw.<br>12 control$.tw.<br>13 or/1-12<br>14 limit 13 to humans<br>15 (hand washing or handwashing or hand hygiene)<br>16 14 and 15<br>17 limit 16 to yr="2009 - Current"<br>18 exp hospitals/<br>19 hospital$.tw.<br>20 exp inpatients/<br>21 inpatient$.tw.<br>22 exp health care/<br>23 health care$.tw.<br>24 healthcare$.tw.<br>25 infirmary$.tw.<br>26 nosocomial$.tw.<br>27 intensive care unit$.tw.<br>28 ward$.tw.<br>29 OR/18-28<br>30 17 and 29<br><br>*EPOC Methodological filter<br>Randomized Controlled Trial [publication type] OR Controlled Clinical Trial [publication type] OR Comparative Study OR Evaluation Studies OR 'comparative study' OR 'effects' OR 'effect' OR 'evaluations' OR 'evaluating' OR 'evaluation' OR 'evaluates' OR 'changing' OR 'changes' OR 'change' OR 'interventions' OR 'intervention' OR 'impact' OR 'random allocation' OR 'post test' OR 'posttest' OR 'pre test' OR 'pretest' OR 'time series' OR 'experimental' OR 'experiments' OR 'experiment' OR 'intervention studies' OR 'intervention study' OR 'controlled clinical trial' OR 'randomised controlled trial' OR 'randomized controlled trial'
EMBASE	1 Handwashing/<br>2 (hand antisepsis or handwash$ or hand wash$ or hand disinfection or hand hygiene or surgical scrub$).tw.<br>3 1 or 2<br>4 exp Hand/<br>5 exp Sterilization/<br>6 4 and 5<br>7 3 or 6	1 Randomized controlled trial/<br>2 random$.tw.<br>3 experiment$.tw.<br>4 (time adj series).tw.<br>5 (pre test or pretest or post test or posttest).tw.<br>6 impact.tw.<br>7 intervention$.tw.<br>8 chang$.tw.<br>9 evaluat$.tw.

	8 randomized controlled trial/<br>9 randomi$.tw.<br>10 exp controlled clinical trial/<br>11 controlled clinical trial$.tw.<br>12 intervention studies/<br>13 experiment$.tw.<br>14 (time adj series).tw.<br>15 (pre test or pretest or (posttest or post test)).tw.<br>16 random allocation/<br>17 impact.tw.<br>18 intervention?.tw.<br>19 chang$.tw.<br>20 evaluation studies/<br>21 evaluat$.tw.<br>22 effect?.tw.<br>23 comparative study/<br>24 animal/<br>25 human/<br>26 24 not 25<br>27 or/8-23<br>28 27 not 26<br>29 7 and 28<br>30 limit 29 to yr="2009 -Current"<br>31 exp hospitals/<br>32 hospital$.tw.<br>33 exp hospital patient/<br>34 inpatient$.tw.<br>35 exp health care/<br>36 health care$.tw.<br>37 healthcare$.tw.<br>38 infirmary$.tw.<br>39 nosocomial$.tw.<br>40 intensive care unit$.tw.<br>41 ward$.tw.<br>42 or/31-41<br>43 30 and 42	10 effect?.tw.<br>11 compar$.tw.<br>12 control$.tw.<br>13 or/1-12<br>14 limit 13 to humans<br>15 (hand washing or handwashing or hand hygiene).<br>16 14 and 15<br>17 limit 16 to yr="2009 - Current"<br>18 exp hospital/<br>19 hospital$.tw.<br>20 exp hospital patient/<br>21 inpatient$.tw.<br>22 exp health care/<br>23 health care$.tw.<br>24 healthcare$.tw.<br>25 infirmary$.tw.<br>26 nosocomial$.tw.<br>27 intensive care unit$.tw.<br>28. ward$.tw.<br>29 or/18-28<br>30 17 and 29
CINAHL	1 (MH "Handwashing+")<br>2 (hand antisepsis OR handwash* OR hand wash* OR hand disinfection OR hand hygiene OR surgical scrub*)<br>3 1 OR 2<br>4 Hand*<br>5 Sterilization*<br>6 4 AND 5<br>7 3 OR 6<br>8 (MH "Clinical Trials+")<br>9 clinical trial*<br>10 randomi*<br>11 controlled clinical trial*<br>12 intervention studies*<br>13 experiment*<br>14 "time series"<br>15 (MH "Pretest-Posttest Design+")<br>16 random allocation*<br>17 impact*<br>18. intervention?	1(MH "Clinical Trials+")<br>2 clinical trial*<br>3 "comparative studies"<br>4 "experimental studies"<br>5 "time series"<br>6 impact*<br>7 evaluat*<br>8 effect*<br>9 (MH "Pretest-Posttest Design+")<br>10 (MH "Quasi-Experimental Studies+")<br>11 or/1-10<br>12 (MH "Handwashing+")<br>13 (handwashing OR hand hygiene)<br>14 or/12-13<br>15 11 and 14<br>16 limit 15 to yr="2009 - Current"<br>17 (MH "Hospitals+")<br>18 (MH "Hospital Units+")<br>19 Intensive Care Units<br>20 (MH "Inpatients")<br>21 (MH "Child, Hospitalized")

	19. chang*<br>20. (MH "Evaluation Research+")<br>21. evaluat*<br>22. effect?<br>23. comparative study*<br>24.(MH "Animals+")<br>25.(MH "Human+")<br>26. 24 NOT 25<br>27. OR/8-23<br>28. 27 NOT 26<br>29. 7 and 28<br>30. limit 29 to yr="2009 -Current"<br>31 (MH "Hospitals+")<br>32 (MH "Hospital Units+")<br>33 hospital*<br>34 Intensive Care Units<br>35 (MH "Inpatients")<br>36 (MH "Child, Hospitalized")<br>37 (MH "Adolescent, Hospitalized")<br>38 (MH "Aged, Hospitalized")<br>39 (hospitalized OR hospitalised)<br>40 (health care OR healthcare)<br>24 healthcare$.tw.<br>25 infirmary$.tw.<br>26 nosocomial$.tw.<br>27 intensive care unit$.tw.<br>28. ward$.tw.<br>41 or/31-40<br>42 30 AND 41	22 (MH "Adolescent, Hospitalized")<br>23 (MH "Aged, Hospitalized")<br>24 (hospitalized OR hospitalised)<br>25 (health care OR healthcare)<br>24 healthcare$.tw.<br>25 infirmary$.tw.<br>26 nosocomial$.tw.<br>27 intensive care unit$.tw.<br>28. ward$.tw.<br>26 or/17-25<br>27 16 AND 26
BNI	1 handwash* (137)<br>2 hand wash* (170)<br>3 hand antisep* (22)<br>4 hand disinfection (39)<br>5 hand hygiene (369)<br>6 hand decontamination (43)<br>7 hand cleansing (29)<br>8 hand cleaning (27)<br>9 1 OR 2 OR 3 OR 4 OR 5 OR 6 OR 7 OR 8 (599)<br>10 hand (1438)<br>11 strilization (106)<br>12 9 OR 11 (702)<br>13 limit 12 to “2009 to Current”	n/a
CRD Database	n/a	1 MeSH DESCRIPTOR Clinical Trial EXPLODE ALL TREES<br>2 Clinical Trial*<br>3 control*<br>4 random*<br>5 comparative stud*<br>6 experimental stud*<br>7 time series*<br>8 impact*<br>9 intervention*<br>10 evaluat*<br>11 effect*<br>12 Chang*<br>13 Compar*<br>14 Experiment*

		15 (pretest OR pre test OR posttest OR post test)<br>16 #1 OR #2 OR #3 OR #4 OR #5 OR #6 OR #7 OR #8 OR #9 OR #10 OR #11 OR #12 OR #13 OR #14 OR #15<br>17 MeSH DESCRIPTOR Handwashing EXPLODE ALL TREES<br>18 (hand washing OR handwashing OR hand hygiene)<br>19 #17 OR #18<br>20 #15 AND #19<br>21 (#20) FROM 2009 TO 2013<br>22 MeSH DESCRIPTOR Hospitals EXPLODE ALL TREES (MH<br>23 MeSH DESCRIPTOR Hospital Units EXPLODE ALL TREES (MH<br>24 hospital*<br>25 Intensive Care Unit*<br>26 MeSH DESCRIPTOR Inpatients EXPLODE ALL TREES<br>27 MeSH DESCRIPTOR Adolescent, Hospitalized EXPLODE ALL TREES<br>28 MeSH DESCRIPTOR Adolescent, Institutionalized EXPLODE ALL TREES<br>29 MeSH DESCRIPTOR Child, Hospitalized EXPLODE ALL TREES<br>30 MeSH DESCRIPTOR Child, Institutionalized EXPLODE ALL TREES<br>31 (hospitalised OR hospitalized OR healthcare OR health care)<br>32 #22 OR #23 OR #24 OR #25 OR #26 OR #27 OR #28 OR #29 OR #30 OR #31<br>33 #21 AND #32
Cochrane Library	1 MeSH descriptor: [Hand hygiene] explode all trees<br>2 (hand antisepsis OR handwash* OR hand wash* OR hand disinfection OR hand hygiene OR surgical scrub*)<br>3 1 OR 2<br>4 Hand*<br>5 Sterilization*<br>6 4 AND 5<br>7 3 OR 6<br>8 MeSH descriptor: [Clinical Trial] explode all trees<br>9 clinical trial*<br>10 randomi*<br>11 controlled clinical trial*<br>12 intervention studies*<br>13 experiment*<br>14 time series*<br>15 (pretest OR pre test OR posttest OR post test)<br>16 random allocation*<br>17 impact*<br>18 intervention?<br>19 chang*<br>20 evaluat*<br>21 effect*	1 MeSH descriptor: [Clinical Trial] explode all trees<br>2 Clinical Trial*<br>3 control*<br>4 random*<br>5 comparative stud*<br>6 experimental stud*<br>7 time series*<br>8 impact*<br>9 intervention*<br>10 evaluat*<br>11 effect*<br>12 Chang*<br>13 Compar*<br>14 Experiment*<br>15 (pretest OR pre test OR posttest OR post test)<br>16 #1 OR #2 OR #3 OR #4 OR #5 OR #6 OR #7 OR #8 OR #9 OR #10 OR #11 OR #12 OR #13 OR #14 OR #15<br>17 MeSH descriptor: [Hand hygiene] explode all trees<br>18 (hand washing OR handwashing OR hand hygiene)<br>19 #17 OR #18<br>20 #16 AND #19

	22 comparative study*<br>23 OR/8-22<br>24 7 and 23<br>25 limit 24 to yr="2009 -Current"<br>26. MeSH descriptor: [Hospitals] explode all trees<br>27 MeSH descriptor: [Hospital Units] explode all trees<br>28 hospital*<br>29 Intensive Care Unit*<br>30 MeSH descriptor: [Inpatients] explode all trees<br>31 MeSH descriptor: [Adolescent, Hospitalized] explode all trees<br>32 MeSH descriptor: [Adolescent, Institutionalized] explode all trees<br>33 MeSH descriptor: [Child, Hospitalized] explode all trees<br>34 MeSH descriptor: [Child, Institutionalized] explode all trees<br>35 (hospitalised OR hospitalized OR healthcare OR health care)<br>36 #26 OR #27 OR #28 OR #29 OR #30 OR #31 OR #32 OR #33 OR #34 OR #35<br>37 #25 AND #36	21 (#20) FROM 2009 TO 2013<br><br>22 MeSH descriptor: [Hospitals] explode all trees<br>23 MeSH descriptor: [Hospital Units] explode all trees<br><br>24 hospital*<br>25 Intensive Care Unit*<br>26 MeSH descriptor: [Inpatients] explode all trees<br>27 MeSH descriptor: [Adolescent, Hospitalized] explode all trees<br>28 MeSH descriptor: [Adolescent, Institutionalized] explode all trees<br>29 MeSH descriptor: [Child, Hospitalized] explode all trees<br>30 MeSH descriptor: [Child, Institutionalized] explode all trees<br>31 (hospitalised OR hospitalized OR healthcare OR health care)<br>32 #22 OR #23 OR #24 OR #25 OR #26 OR #27 OR #28 OR #29 OR #30 OR #31<br>32 #21 AND #32
Current Clinical Control Trial	n/a	("hand hygiene" OR "hand washing" OR "handwashing" OR "hand sanitizer" OR "hand rubbing" OR "hand rubs") AND ("hospital" OR "healthcare" OR "inpatients" OR "intensive care unit" OR "hospitalised" OR "hospitalized" OR “nosocomial”)
ACP journal	("hand hygiene" OR "hand washing" OR "handwashing" OR "hand sanitizer" OR "hand rubbing" OR "hand rubs") AND ("hospital" OR "healthcare" OR "inpatients" OR "intensive care unit" OR "hospitalised" OR "hospitalized" OR “nosocomial”)	n/a
Evidence-Based Medicine Reviews	1 handwashing.sh.<br>2 handwash$.tx.<br>3 hand wash$.tx.<br>4 hand disinfection.tx.<br>5 hand hygiene.tx.<br>6 surgical scrub$.tx.<br>7 hand decontamination.mp. [mp=ti, to, ab, tx, kw, ct, sh, hw]<br>8 hand cleansing.mp. [mp=ti, to, ab, tx, kw, ct, sh, hw]<br>9 hand cleaning.mp. [mp=ti, to, ab, tx, kw, ct, sh, hw]<br>10 1or2or3or4or5or6or7or8or9<br>11 from 10 keep 1-249<br>12 10<br>13 limit 12 to yr=”2005 Current”	n/a
