# Supplementary material for: Comparative efficacy of interventions to promote hand hygiene in hospital: systematic review and network meta-analysis
Source: BMJ. 2015 Jul 28;351:h3728. doi: 10.1136/bmj.h3728 (PMC4517539; doi:10.1136/bmj.h3728)
Supplement: Supplementary file 2 [file luan024450.ww2_default.pdf]

Appendix 2: Classification for level of information on resources use [posted as supplied by author]

Level of information on resources use for interventions

High: Stated clearly what the interventions were and how they were implemented and described clearly what materials were used and how much time for each person was spent as well as the duration of the implementation period.

Moderate: Stated what the interventions were and how they were implemented but lacking a clear description of materials used and person-time involved as well as time spent for each intervention.

Low: Stated only what the interventions were and how they were implemented. Largely lacking any details on materials used, person-time involved as well as the time spent for each intervention.
