# Supplementary material for: Comparative efficacy of interventions to promote hand hygiene in hospital: systematic review and network meta-analysis
Source: BMJ. 2015 Jul 28;351:h3728. doi: 10.1136/bmj.h3728 (PMC4517539; doi:10.1136/bmj.h3728)
Supplement: Supplementary file 3 [file luan024450.ww3_default.pdf]

## Appendix 3: Analysis of interrupted time series data [posted as supplied by author]

Data from interrupted time series were re-analysed when data on the number of opportunities and hand hygiene compliance at different time points could be obtained[1]. We used a segmented logistic regression model[2].

If $n(t)$ represents the number of hand hygiene opportunities in a study at time $t$ and $y(t)$ represents the number of occasions where compliance was observed, then we used the following generalized linear model to evaluate the effect of the intervention:

$$y(t) \sim \text{binomial}(\pi(t), n(t)) \qquad [1]$$

$$\ln(\pi(t)/(1-\pi(t))= a + b\times t + c\times\mathbf{1}_{t\geq t.int} + d\times\mathbf{1}_{t\geq t.int}\times(t-t.int) \qquad [2]$$

where $\pi(t)$ is the probability of hand hygiene at time $t$, $t.int$ is the time of the intervention, $\mathbf{1}_{t\geq t.int}$ is a function of $t$ taking the value 1 if $t\geq t.int$ and zero otherwise. In this expression the parameter $a$ measures baseline compliance, $b$ the initial pre-intervention trend, $c$ the step (level) change associated with the intervention, and $d$ corresponds to the change in trend associated with the intervention. These parameters were estimated for each study that was re-analysed. In this model an intervention can increase hand hygiene either through a step increase in compliance at the time of the intervention ($c > 0$) or through a trend for increased compliance ($d > 0$).

It is also useful to obtain a statistic that summarizes the effectiveness of the intervention, accounting for both changes in trend and level. There are several possibilities and we consider two: the mean percentage change in hand hygiene compliance in the post-intervention period attributed to the intervention (an absolute measure of change in compliance) and the mean log odds ratio of hand hygiene associated with the intervention (a relative measure).

The first statistic, the mean percentage change in hand hygiene compliance, is given by 100 times the mean difference between the value of $\pi(t)$ predicted by equation [2] and the value of $\pi(t)$ that would be expected if the terms $c$ and $d$ were set to zero (i.e. the expected compliance probability if the intervention had not occurred), where the mean is taken over the post-intervention interval [$t.int$, $t.end$], where $t.end$ is the time of the end of post-intervention period. This is equivalent to $100/(t.end - t.int)$ multiplied by the area between the following two curves (representing the hand hygiene compliance probability given the intervention and the hand hygiene compliance probability that would be expected without the intervention, respectively) for $t.int \leq t \leq t.end$:

$$\pi_1(t) = \frac{exp(a + bt + c1_{t\geq t.int} + d1_{t\geq t.int}(t - t.int))}{1 + exp(a + bt + c1_{t\geq t.int} + d1_{t\geq t.int}(t - t.int))} \qquad [3]$$

$$\pi_0(t) = \frac{exp(a + bt)}{1 + exp(a + bt)} \qquad [4]$$

[1] If only the total number of hygiene opportunities was reported it was assumed that these opportunities were equally distributed amongst the different observation periods.

[2] Taljaard M, McKenzie JE, Ramsay CR, Grimshaw JM (2014). The use of segmented regression in analysing interrupted time series studies: an example in pre-hospital ambulance care. Implement Sci. 9:77.

This is given by $100 \times \frac{A_1 - A_0}{t.end - t.int}$ where the areas $A_1$ and $A_2$ are found by integrating [3] and [4] over this range:

$$A_1 = \int_{t.int}^{t.end} \pi_1(t)dt \qquad\qquad A_0 = \int_{t.int}^{t.end} \pi_0(t)dt$$

which gives

$$A_1 = \frac{\ln(1+\exp(a+b\times t.end+c+d\times(t.end-t.int))}{(b+d)(1+\exp(a+b\times t.int+c))}$$

$$A_0 = \frac{\ln(1+\exp(a+b\times t.end))}{b(1+\exp(a+b\times t.int))}.$$

An associated standard error was obtained using the delta method making use of the covariance matrix obtained by fitting the full generalized linear model described by equations [1] and [2].

The relative measure of hand hygiene change associated with the intervention is the mean log odds ratio for hand hygiene. This is defined as the mean value of the logarithm of the ratio of the odds of hand hygiene compliance in the post-intervention period given by equation [2] to the odds of hand hygiene given by equation [2] but setting term $c$ and $d$ to zero. This is given by

$$c + d \times (t.end - t.int)/2$$

and the associated variance is given by

$$\mathrm{var}(c) + \mathrm{var}(d) \times ((t.end - t.int)/2)^2 + 2 \times \mathrm{cov}(c, d) \times (t.end - t.int)/2.$$
