# Supplementary material for: Comparative efficacy of interventions to promote hand hygiene in hospital: systematic review and network meta-analysis
Source: BMJ. 2015 Jul 28;351:h3728. doi: 10.1136/bmj.h3728 (PMC4517539; doi:10.1136/bmj.h3728)
Supplement: Supplementary file 4 [file luan024450.ww4_default.pdf]

Appendix 4: WINBUGs code for network meta-analysis [posted as supplied by author]

a) Base case analysis

```
# Trial-level data given as treatment differences
# Random effects model for multi-arm trials
model{                                 # *** PROGRAM STARTS
   for(i in 1:ns) {                    # LOOP THROUGH STUDIES
   y[i] ~ dnorm(delta[i],prec[i])      # normal likelihood for trials
   var[i] <- pow(se[i],2)              # calculate variances
   prec[i] <- 1/var[i]                 # set precisions

   # Trial-specific meandiff distributions
   delta[i] ~ dnorm(md[i],tau)

   #Mean of random effects distributions,with multi-arm trial correct
   md[i] <-  d[t[i,2]] - d[t[i,1]]

   #Deviance contribution
   dev[i] <- (y[i]-delta[i])*(y[i]-delta[i])*prec[i]

   #summed residual deviance contribution for this trial
   resdev[i] <- sum(dev[i])
 }
      totresdev <- sum(resdev[])            #Total Residual Deviance
      d[1]<-0       # Treatment effect is zero for reference treatment
                    # Vague priors for treatment effects
      for (k in 2:nt){d[k] ~ dnorm(0,.0001)
     }
      between.trial.var ~ dunif(0,5)
      tau<- 1/between.trial.var

       test~dnorm(2,4)I(0.1,)

       #Ranking
       for (k in 1:4) {
       rk[k] <- 5-rank(d[],k)
       best[k] <- equals(rk[k],1)}
}                                       # *** PROGRAM ENDS

# ns= number of studies;
# nt=number of treatments;

list(ns=12, nt=4, nc=6)
c[]   t[,1] t[,2] y[]   se[]
1     1     3     0.4711      0.1647
2     1     4     2.4499      0.1691
3     2     4     0.7974      0.4078
4     4     4     1.0656      0.1131
2     1     4     2.2700      0.3041
1     1     3     5.4996      1.4148
1     1     3     1.9448      0.8245
2     1     4     2.8740      0.1402
1     1     3     1.3230      0.8183
5     3     3     -1.8738     0.6183
6     2     3     0.6408      0.4910
5     3     3     -0.5222     1.2273
END
```

Dbar = post.mean of -2logL; Dhat = -2LogL at post.mean of stochastic nodes

	Dbar	Dhat	pD	DIC
test	0.000	0.000	-0.000	0.000
y	13.467	3.074	10.396	23.860
total	13.467	3.074	10.396	23.860

b) Sensitivity analysis

Code and results for sensitivity analysis excluding studies where interventions were introduced at multiple time points (Helms et al., Kirkland et al., and Al-Tawfiq et al).

```
# Trial-level data given as treatment differences
# Random effects model for multi-arm trials
model{                                 # *** PROGRAM STARTS
   for(i in 1:ns) {                    # LOOP THROUGH STUDIES
   y[i] ~ dnorm(delta[i],prec[i])      # normal likelihood for trials
   var[i] <- pow(se[i],2)              # calculate variances
   prec[i] <- 1/var[i]                 # set precisions

   # Trial-specific meandiff distributions
      delta[i] ~ dnorm(md[i],tau)

   #Mean of random effects distributions,with multi-arm trial correct
   md[i] <-  d[t[i,2]] - d[t[i,1]]

   #Deviance contribution
   dev[i] <- (y[i]-delta[i])*(y[i]-delta[i])*prec[i]

   #summed residual deviance contribution for this trial
   resdev[i] <- sum(dev[i])
 }
      totresdev <- sum(resdev[])            #Total Residual Deviance
      d[1]<-0      # Treatment effect is zero for reference treatment
                   # Vague priors for treatment effects
      for (k in 2:nt){d[k] ~ dnorm(0,.0001)
     }
      between.trial.var ~ dunif(0,5)
      tau<- 1/between.trial.var

       test~dnorm(2,4)I(0.1,)

       #Ranking
       for (k in 1:4) {
       rk[k] <- 5-rank(d[],k)
       best[k] <- equals(rk[k],1)}
}                                       # *** PROGRAM ENDS

# ns= number of studies;
# nt=number of treatments;



list(ns=9, nt=4, nc=6)
c[]   t[,1] t[,2] y[]   se[]
1     1     3     0.4711      0.1647
3     2     4     0.7974      0.4078
4     4     4     1.0656      0.1131
2     1     4     2.2700      0.3041
2     1     4     2.8740      0.1402
1     1     3     1.3230      0.8183
5     3     3     -1.8738     0.6183
6     2     3     0.6408      0.4910
5     3     3     -0.5222     1.2273
END
```

Dbar = post.mean of -2logL; Dhat = -2LogL at post.mean of stochastic nodes

	**Dbar**	**Dhat**	**pD**	**DIC**
test	0.000	0.000	-0.000	0.000
y	6.661	-1.189	7.851	14.512
total	6.661	-1.189	7.851	14.512
