# Supplementary material for: Comparative efficacy of interventions to promote hand hygiene in hospital: systematic review and network meta-analysis
Source: BMJ. 2015 Jul 28;351:h3728. doi: 10.1136/bmj.h3728 (PMC4517539; doi:10.1136/bmj.h3728)
Supplement: Supplementary file 5 [file luan024450.ww5_default.pdf]

Appendix 5: Excluded studies with reason by EPOC criteria [posted as supplied by author]

No.	Authors	Year	Meet inclusion criteria	Meet EPOC criteria	Reason for exclusion [a]
1	Abela	2012	Y	N	UBA with no control
2	Aboumatar	2012	Y	N	ITS with inadequate data collection points
3	Alemagno	2010	Y	N	UBA with no control
4	Allegranzi	2010	Y	N	UBA with no control
5	Allegranzi	2013	Y	N	UBA with no control
6	Ananda-Rajah	2010	Y	N	ITS with inadequate data collection points
7	Apisarnthanarak	2010a	Y	N	UBA with no control
8	Apisarnthanarak	2010b	Y	N	CBA with uncomparable control
9	Ardizzone	2013	Y	N	UBA with no control
10	Barahona-Guzman	2014	Y	N	ITS with inadequate data collection points
11	Barbut	2013	Y	N	UBA for AHR use/ ITS for HAI rate
12	Barrera	2011	Y	N	ITS with inadequate data collection points
13	Bessesen	2013	Y	N	UBA with no control
14	Bingham	2010	Y	N	UBA with no control
15	Biswal	2013	Y	N	UBA with no control
16	Boog	2013	Y	N	ITS with inadequate data collection points
17	Borges	2012	Y	N	UBA with no control
18	Bouadma	2010	Y	N	UBA with no control
19	Buffet-Bataillon	2010	Y	N	UBA with no control
20	Bukhari	2011	Y	N	UBA, no baseline data
21	Caniza	2009	Y	N	UBA with no control
22	Chen	2011	Y	N	UBA with no control
23	Cheng	2010	Y	N	UBA for AHR use/ ITS for HAI rate
24	Costers	2012	Y	N	UBA with no control
25	Cumbler	2013	Y	N	ITS with inadequate data collection points
26	Davis	2010	Y	N	UBA with no control
27	Macedo	2012	Y	N	UBA with no control
28	di Martino	2011	Y	N	UBA with no control
29	DiDiodato	2013	Y	N	UBA with no control
30	Dierssen-Sotos	2010a	Y	N	UBA with no control
31	Dierssen-Sotos	2010b	Y	N	UBA with no control
32	Dilek	2012	Y	N	UBA with no control

33	Dos Santos	2013	Y	N	ITS with inadequate data collection points
34	El-Kafrawy	2013	Y	N	UBA with no control
35	Eveillard	2011	Y	N	UBA with no control
36	Fakhry	2012	Y	N	UBA with no control
37	Fitzpatrick	2011	Y	N	UBA with no control
38	Forrester	2010	Y	N	UBA with no control
39	Garcia-Rodriguez	2013	Y	N	UBA with no control
40	Garcia-Vazquez	2011	Y	N	UBA with no control
41	Gill	2009	Y	N	CBA with only 1 control
42	Graf	2013	Y	N	UBA with no control
43	Grant	2011	Y	N	UBA with no control
44	Grayson	2011	Y	N	ITS with inadequate data collection points
45	Harne-Britner	2011	Y	N	CBA with only 1 control
46	Helder	2010	Y	N	UBA with no control
47	Helder	2012	Y	N	UBA, no baseline data
48	Henderson	2012	Y	N	UBA with no control
49	Homa	2011	Y	N	ITS with inadequate data collection points
50	Jaggi	2013	Y	N	UBA with no control
51	Jamal	2012	Y	N	ITS with inadequate data collection points
52	Jeong	2013	Y	N	UBA with no control
53	Kanj	2013	Y	N	UBA with no control
54	KelcÃkova	2012	Y	N	UBA, no baseline data
55	Kim	2013	Y	N	UBA with no control
56	Kindness	2010	Y	N	UBA, no baseline data
57	Kowitt	2013	Y	N	ITS with inadequate data collection points
58	Langston	2011	Y	N	UBA with no control
59	Leblebicioglu	2013a	Y	N	UBA with no control
60	Leblebicioglu	2013b	Y	N	UBA with no control
61	Levchenko	2011	Y	N	UBA with no control
62	Linam	2011	Y	N	UBA with no control
63	Ling	2012	Y	N	UBA with no control
64	Lobo	2010	Y	N	UBA with no control
65	Marra	2013	Y	N	UBA, no baseline data
66	Mathai	2011	Y	N	UBA with no control
67	Mazi	2013	Y	N	ITS with inadequate data collection points
68	Molina-Cabrillana	2010	Y	N	UBA with no control
69	Monistrol	2012	Y	N	UBA with no control
70	Monistrol	2013	Y	N	UBA with no control
71	Mukerji	2013	Y	N	UBA with no control
72	Pontivivo	2012	Y	N	ITS with inadequate data collection points

73	Prospero	2010	Y	N	UBA with no control
74	Rahim	2009	Y	N	UBA with no control
75	Rees	2013	Y	N	ITS with inadequate data collection points
76	Reichardt	2014	Y	N	UBA with no control
77	Rello	2013	Y	N	UBA with no control
78	Roberts	2012	Y	N	ITS with inadequate data collection points
79	Rogers	2010	Y	N	UBA with no control
80	Rosenthal	2010	Y	N	UBA with no control
81	Rosenthal	2012a	Y	N	UBA with no control
82	Rosenthal	2012b	Y	N	UBA with no control
83	Rosenthal	2012c	Y	N	UBA with no control
84	Rosenthal	2012d	Y	N	UBA with no control
85	Rosenthal	2013	Y	N	UBA with no control
86	Sahud	2012	Y	N	ITS with inadequate data collection points
87	Saint	2009	Y	N	UBA with no control
88	Salama	2013	Y	N	UBA with no control
89	Santos	2013	Y	N	UBA with no control
90	Saramma	2011	Y	N	UBA with no control
91	Scheithauer	2012	Y	N	UBA, no baseline data
92	Scheithauer	2013a	Y	N	UBA with no control
93	Scheithauer	2013b	Y	N	UBA with no control
94	Scheithauer	2013c	Y	N	UBA with no control
95	Seirafian	2013	Y	N	UBA with no control
96	Seto	2013	Y	N	ITS with inadequate data collection points
97	Simmons	2013	Y	N	UBA with no control
98	Son	2011	Y	N	UBA with no control
99	Song	2013	Y	N	ITS with inadequate data collection points
100	Tromp	2012	Y	N	UBA with no control
101	van den Hoogen	2011	Y	N	UBA with no control

[a] UBA, uncontrolled before-after study; CBA, controlled before-after study; ITS, interrupted time series study; AHR, alcohol based hand rub; and HAI, healthcare associated infection.
