# Supplementary material for: Comparative efficacy of interventions to promote hand hygiene in hospital: systematic review and network meta-analysis
Source: BMJ. 2015 Jul 28;351:h3728. doi: 10.1136/bmj.h3728 (PMC4517539; doi:10.1136/bmj.h3728)
Supplement: Supplementary file 6 [file luan024450.ww6_default.pdf]

Appendix 6: Summary of study characteristics. [posted as supplied by author]

Author (year)	Study design	Study period/ duration	Settings and Details of the design	Unit of Participant	Population	Interventions	Comparison group	Methods of observation	Indication	Outcomes	Country
Fuller (2012)	CRCT (Stepped wedge)	Total: 39 months<br>Baseline: approx. 20 months<br>Intervention: approx. 20 months<br><br>October 2006 to December 2009	60 wards from 16 hospital trusts<br><br>Experimental: 33 mixed wards from 13 trusts<br>Control: 27 mixed wards from 11 trusts	Ward	HCWs	WHO-5+GOAL+FED	WHO-5 (UK national campaign)	Direct observation by ward coordinators	Before and after patient contact	Hand hygiene compliance<br>Soap and AHR consumption	England and Wales
Huis (2013)	CRCT	Total: 13 months<br>Baseline: 1 month<br>Intervention: 6 months<br>Follow up (no intervention): 6 months<br><br>September 2008 to November 2009	67 wards from 3 hospitals<br><br>Experimental: 30 mixed wards<br>Control: 37 mixed wards	Ward	Nurses	WHO-5+GOAL	WHO-5 (except SAF)	Direct observation by nursing student	Before and after patient contact, patient surroundings and use of gloves	Hand hygiene compliance	Netherlands
Mertz (2010)	CRCT	Total: 15 months<br>Baseline: 3 months<br>Intervention: 12 months<br><br>October 2006 to December 2006 (Baseline) and June 2007 to May 2008 (Intervention)	30 wards from 3 acute care sites<br><br>Experimental: 15 mixed wards<br>Control: 15 mixed wards<br><br>Randomized stratified by hospital site and type of hospital unit.<br>(number of bed not reported)	Ward	HCWs	SYS+EDU+FED +REM	SYS (with AHR at point of care)	Direct observation by researchers	Before and after patient contact, skin wounds or mucous, insertion of an intravenous line and use of gloves	Hand hygiene compliance<br>Incidence of hospital-acquired MRSA colonisation	Canada

Huang (2002)	RCT	Intervention: 4 months period (Pre and Post-test)<br><br>September 2000 to January 2001	100 randomly selected nurses from a 1,300 bed hospital<br><br>Experimental: 50 nurses<br>Control: 50 nurses	Individual	Nurses	EDU	None (unclear AHR)	Direct observation by researchers	Before and after patient contact	Hand washing compliance	China
Fisher (2013)	RCT	Total: 24 weeks<br>Baseline: 14 weeks<br>Intervention I: 6 week<br>Intervention II: 4 weeks<br><br>Start from January 2012	233 participants from 3 wards from 2 hospitals<br><br>Experimental: 119 participants<br>Control: 114 participants	Individual	HCWs	SYS+REM+FED	SYS (with AHR at point of care)	Direct observation by trained nurses	Room entry and exit	Hand hygiene compliance<br>AHR consumption	Singapore
Salamati (2013)	RCT	No data provided<br><br>Year 2010	128 participants from a 109-bed hospital<br><br>Experimental: 64 participants<br>Control: 64 participants	Individual	Nurses, Anesthesiology, Technician, and Nurese-aid	SYS+EDU+FED	SYS+EDU	Direct observation by infection control supervisor	Unclear	Hand hygiene score with unclear details	Iran
Derde (2014)	ITS	Total: 36 months (3 years)<br>Baseline: 6 months<br>Intervention I: 18 months<br>Intervention II: 12 months<br><br>May 2008 to April 2011	13 European ICUs (all with least 8 beds)	Ward	HCWs	EDU+FED+REM	REM (unclear AHR use)	Direct observation by researchers	Before and after patient contact, before an aseptic task, and after contact with patients' body fluids and surroundings	Hand hygiene compliance<br>Acquisition of MRSA, vancomycin-resistant *enterococci* (*VRE*), and highly resistant *Enterobacteriaceae* (*HRE*).	Multiple European countries

Lee (2013)	ITS	Total: 24 months (2 years)<br>Baseline: 6 months<br>Intervention: 12 months<br>Post-Intervention (Wash out): 6 months<br><br>March 2008 to July 2010	33 Surgical wards of 10 hospitals in 9 countries	Ward	HCWs	WHO-5	None (with AHR) in 1 hospital,<br>SYS in 1 hospital and WHO-5 in 2 hospitals	Direct observation by researchers	Before and after patient contact, before an aseptic task, and after contact patients' body fluids and surroundings	Hand hygiene compliance<br>MRSA infection rates<br>MRSA surgical site infection rates	Multi-center<br>9 European countries including Germany, Israel, Serbia, Switzerland, France, Greece, Italy, Spain, Scotland
Marra (2013)	ITS	Total: 12 months<br>Baseline: 3 months<br>Intervention: 9 months<br><br>August 2011 to July 2012	9 wards (8 ICUs and 1 general ward)<br>from 7 tertiary hospitals in 2 countries	Ward	HCWs	WHO-5	None (with AHR)	Direct observation by trained nurses	WHO "Five Moments of Hand Hygiene"	Hand hygiene compliance<br>Device-associated infection rate	Brazil and Thailand
Al-Tawfiq (2013)	ITS	Total: 54 months (4 years 6 months)<br>Baseline: 9 months<br>Intervention: 42 months (implemented at multiple time points)<br>Post-Intervention: 3 months<br><br>October 2006 to December 2011	A 350-bed hospital	Hospital	HCWs	WHO-5+GOAL	None (with AHR)	Direct observation by infection control team	Before wearing gloves, after removing gloves, before and after patient contact, after leaving patient's room, before and after performing invasive procedures, and after contact with patient's	Hand hygiene compliance<br>AHR consumption<br>Healthcare-associated MRSA, Ventilator-associated infection, Central line-associated bloodstream infections and Catheter-associated urinary tract infection.	Saudi Arabia

									body fluids.		
Armellino (2013)	ITS	Total: 17 months<br>Baseline: 1 month<br>Intervention: 16 months<br><br>March 2010 to July 2011	An 18-bed surgical intensive care unit (SICU) from a 804-bed hospital	Ward	HCWs	FED + GOAL	None (unclear AHR use)	Electronic motion sensor and video recorders and sinks and dispensers. Review and audit by researchers	Room entry and exit	Hand hygiene compliance	USA
Armellino (2012)	ITS	Total: 25 months<br>Baseline: 4 months<br>Intervention: 4 months<br>Maintenance: 17 months<br><br>June 2008 to June 2010	A 17-bed medical intensive care unit from a 804-bed hospital	Ward	HCWs	FED + GOAL	None (unclear AHR use)	Electronic motion sensor and video recorders and sinks and dispensers. Review and audit by researchers	Room entry and exit	Hand hygiene compliance	USA
Chan (2013)	ITS	Total: 7 months<br>Baseline: 1 month<br>Intervention: 6 months<br><br>April 2009 to October 2009	A general medical unit with 10 single rooms	Ward	HCWs and others including patients and relatives	SYS	None (with AHR)	n/a	Dispenser count	Hand hygiene event per patient-day	USA
Crews (2013)	ITS	Total: 63 months (5 years 3 months)<br>Baseline: 9 months<br>Intervention: 15 months (implemented at multiple time points)<br>Post-Intervention: 39 months	A 46-bed children hospital	Hospital	HCWs	SYS+EDU+FED +REM+ INC+GOAL	EDU (with AHR)	Direct observationby contracted certified infection control practitioner	Before and after contact patient or environment	Hand hygiene compliance	USA

		October 2006 to December 2011									
Salmon (2013)	ITS	Total: 45 months (3 years 9 months)<br>Baseline: 18 months<br>Intervention: 3 month<br>Post-Intervention: 24 months<br><br>January 2009 to September 2012	A 1,032-bed hospital	Hospital	Nursing students	EDU	None (unclear AHR use)	Direct observation by infection control nurses	WHO "Five Moments of Hand Hygiene"	Hand hygiene compliance	Singapore
Talbot (2013)	ITS	Total: 68 months (5 years 8 months)<br>Baseline: 29 months<br>Intervention phase I: 18 months<br>Intervention phase II: 21 months<br><br>January 2007 to August 2012	A university medical center (support 65,000 inpatient admission, annually)	Hospital	HCWs<br>Settings were inpatient, outpatient clinics and procedural areas	Phase I: WHO-5 +INC+GOAL<br>Phase II: WHO-5 +INC+GOAL+ACC	Phase I: EDU (unclear AHR use)<br>Phase II: WHO-5+INC+GOAL	Direct observation by trained healthcare workers	WHO "Five Moments of Hand Hygiene"	Hand hygiene compliance<br>Device-associated infection rate	USA
Higgins (2013)	ITS	Total: 30 months (2 years 6 months)<br>Baseline: 15 months<br>Intervention: 15 months<br><br>November 2009 to April 2012	A tertiary referral private hospital<br>(acute healthcare setting)<br><br>(number of bed not reported)	Hospital	HCWs	WHO-5+INC	None (with AHR)	Direct observation by trained infection prevention control nurses	WHO "Five Moments of Hand Hygiene"	Hand hygiene compliance	Ireland
Helder (2012)	ITS (for hand hygiene event)	Total: 4 months<br>Baseline: 2 months<br>Intervention: 2 months	A 27-bed Neonatal Intensive Care Unit (NICU)	Ward	HCWs	(SYS+) REM	SYS (with AHR at POC)	Electronic dispensers counting<br>Direct	Before and after touching a patient, before sterile	Hand hygiene compliance (pre-post test)<br>Hand hygiene event	Netherlands

	BA (for HHC% observation) not eligible as no control group and inadequate data collection point	January 2008 to May 2008						observation by medical students	procedures, before and after the use of gloves and after contact with body fluids	per patient-day	
Kirkland (2012)	ITS	Total: 48 months (4 years)<br>Baseline: 6 months<br>Intervention: 42 months<br><br>January 2006 to November 2009	A 383-bed teaching hospital	Hospital	HCWs	WHO-5	None (with AHR)	Direct observation by trained infection control staff	Before and after contact with patients or their immediate environment	Hand hygiene compliance<br>*Staphylococcus aureus* infections, *Clostridium difficile* infections and bloodstream infections	USA
Morgan (2012)	ITS	Total: 30 weeks<br>Baseline: 15 weeks<br>Intervention: 15 weeks<br><br>March 2010 to October 2010	2 ITS wards; Neurological ICU and Cardiac ICU, 15 beds each	Ward	HCWs	SYS+EDU+FED +REM	None (with AHR)	Direct observation by trained researcher	Room entry and exit	Hand hygiene compliance	USA
Stone (2012)	ITS	Total: 48 months (4 years)<br>Baseline: 5 months<br>Intervention: 43 months<br><br>July 2004 to June 2008	187 acute hospital trusts	Trust (Hospital)	HCWs	WHO-5	None (unclear AHR use)	n/a	Procurement	AHR consumption<br>Antimicrobial consumption<br>Incidence of hospital acquired MRSA and MSSA bacteraemia	England and Wales

Jaggi (2012)	ITS	Total: 12 months<br>Baseline: 6 months<br>Intervention: 6 months<br><br>January 2009 to December 2009	A 215-bed tertiary-care hospital	Hospital	HCWs	Unclear	Unclear	Direct observation by staff	Unclear	Hand hygiene compliance<br>Central line-associated bloodstream infection rate (CLABSI)	India
Lee (2012)	ITS	Total: 6 years<br>Baseline: 3 years<br>Intervention: 3 years<br><br>January 2004 to December 2010	A 1162-bed tertiary-care university hospital	Hospital	HCWs	WHO-5	None (with AHR)	n/a	Procurement	AHR consumption<br>Antimicrobial consumption<br>Incidence of healthcare acquired infection and hospital acquired-MRSA	Taiwan
Mestre (2012)	ITS	Total: 51 months (4 years 3 months)<br>Baseline: 27 months<br>Intervention phase I: 12 months (2010)<br>Intervention phase II: 12 months (2011)<br><br>March 2007 to December 2011	A private 200-bed hospital	Hospital	HCWs	Phase I: WHO-5<br>Phase II: WHO-5 (intense) + Reinforcement	Phase I: None (with AHR)<br>Phase II: WHO-5	Direct observation by infection control and nursing supervisors	WHO "Five Moments of Hand Hygiene"	Hand hygiene compliance<br><br>AHR consumption<br><br>Hospital-acquired MRSA infection rate	Spain
Koff (2011)	ITS	Total: 36 months<br>Baseline: 12 month<br>Intervention: 12 months<br>Post-Intervention: 12 months<br><br>December 2006 to	A medical-surgical ICU<br><br>(number of beds not reported)	Ward	HCWs (nursing staff, physicians, and respiratory therapists)	EDU+FED	None (with AHR)	Direct observation by infection control	Upon entering the patient care environment and after leaving	Hand hygiene compliance<br>Hand hygiene decontamination event<br>Ventilator associated pneumonia (VAP)	USA

		November 2009								rate<br>Catheter-related bloodstream infection (CRBSI) rate	
Doron (2011)	ITS	Total: 18 months<br>Baseline: 6 months<br>Intervention: 12 months<br><br>September 2007 to February 2009	A 425-bed hospital, an academic medical center	Hospital	HCWs	WHO-5	SYS+EDU+FED+REM (with AHR)	Direct observation by trained staff	Before touching the patient or an object in the patient's room and before or after the encounter with the patient	Hand hygiene compliance	USA
Marra (2010, 2011)	ITS	Total: 21 months,<br>East Step-down Unit (SDU)<br>Baseline: 3 months<br>Intervention: 19 months<br><br>West Step-down Unit (SDU)<br>Baseline: 6 months<br>Intervention: 15 months<br><br>April 2008 to November 2009	Two 20-bed SDUs from a private tertiary care hospital (all had single bed rooms)	Ward	HCWs	WHO-5	None (with AHR)	n/a	Dispenser count	Hand hygiene event<br>Alcohol gel consumption<br>Incidence of healthcare associated infections	Brazil
Yngstrom (2011)	ITS	Total: 10 months<br>Baseline: 3 months<br>Intervention: 6 months<br><br>September 2004 to	A 110-bed multidisciplinary district hospital	Hospital	HCWs	SYS+EDU+FED+GOAL	None (with AHR)	Direct observation by trained nurse at each ward	Use of short-sleeved uniforms, protective clothing, aprons and gloves,	Basic hygiene compliance<br>Incidence of healthcare associated infection in ventilated	Sweden

		June 2005							hand disinfection with AHR, and wearing rings and wristwatches	patients (healthcare-associated infections with regard to ventilator associated pneumonia, intubation-related infections in blood vessels and healthcare-associated urinary infections).	
Helms (2010)	ITS	Total: 12 months<br>Baseline: 3 months<br>Intervention: 9 months<br><br>August 2007 to July 2008	A 116-bed hospital	Hospital	HCWs	WHO-5	None (with AHR)	Direct observation by selected staff members and volunteers	WHO "Five Moments of Hand Hygiene" *(Information from author contact)	Hand hygiene compliance<br>Incidence of HAIs; urinary tract infection, ventilator associated pneumonia and central line infections	USA
Chou (2010)	ITS	Total: 57 months (4 years 9 months)<br>Baseline: 21 months<br>Intervention: 36 months<br><br>April 2005 to December 2009	A hospital, part of a 9-hospital healthcare system	Hospital	HCWs	WHO-5+INC+GOAL	None (with AHR)	Direct observation by a staff liaison from each department	Based on opportunities	Hand hygiene compliance	USA

Vernaz (2008)	ITS	Total: 72 months (6 years)<br>Baseline: 22 months (1 year and 10 months)<br>Intervention: 50 months<br>(4 years and 2 months)<br><br>February 2000 to September 2006	A 2,200 bed tertiary university hospital	Hospital	HCWs	WHO-5 (Swiss national campaign)	SYS (with AHR at point of care)	n/a	Procurement	AHR consumption<br>Antibiotics use<br>Incidence of MRSA and *C.difficile* (number of clinical isolates per 100 patient-days)	Switzerland
Whitby (2008)	ITS	Total: 2 years<br>Baseline: 4 to 5 months<br>Intervention: 18 months<br><br>2004 to 2006	5 wards from a 800-bed hospital<br>with 3 intervention groups<br>1) Washington campaign (1 ward)<br>2) Geneva campaign (2 wards)<br>3) AHR substitution (2 wards)	Ward	HCWs	Group I: WHO-5<br>Group II: SYS+EDU+REM +SAF<br>Group III: SYS	None (with AHR)	n/a	Dispenser (liquid soap) count	Hand hygiene events per occupied bed-day	Australia
Grayson (2008)	ITS	Total: 48 months<br>Baseline: 24 months<br>Intervention: 24 months<br><br>October 2002 to September 2006	6 healthcare institutions<br>4 urban sites (403 to 632 beds) and<br>2 regional sites (120 and 271 beds)	Hospital	HCWs	WHO-5 (Hand Hygiene Culture Change Program)	None	Direct observation by trained HCWs	WHO "Five Moments of Hand Hygiene"	Hand hygiene compliance<br><br>AHR consumption<br><br>MRSA bacteraemia and clinical isolates	Australia
Eldridge (2006)	ITS	Total: 15 months<br>Baseline: 4 and 6 months<br>Intervention: 11 and 9 months<br><br>July 2003 to	4 intensive care units from 3 hospitals	Ward	HCWs	WHO-5 (CDC's guidelines)	None	Direct observation by nursing staff	Based on opportunities	Hand hygiene compliance<br><br>AHR consumption	USA

		September 2004									
Johnson (2005)	ITS	Total: 64 months<br>Baseline: 28 months<br>Intervention: 36 months<br><br>January 1999 to April 2004	A 840-bed university teaching hospital	Hospital	HCWs	WHO-5 (Hand Hygiene Culture Change Program)	None	Direct observation by trained HCWs	WHO "Five Moments of Hand Hygiene"	Hand hygiene compliance<br><br>AHR consumption<br><br>MRSA bacteraemia and clinical isolates of ESBL-producing *E coli* and *Klebsiella spp.*	Australia
Khatib (1999)	ITS	Total: 8 weeks<br>Baseline: 4 weeks<br>Intervention: 4 weeks<br><br>Data on date is not available	One ICU	Ward	Respiratory care practitioner	EDU+REM	EDU	Direct observation by nursing staff	Before and after mechanically ventilated patient contact	Hand hygiene compliance (hand washing)	USA
Tibballs (1996)	ITS	Total: 25 weeks (5 months)<br>Baseline: 9 weeks<br>Intervention: 4 weeks<br>Postintervention: 12 weeks<br><br>Data on date is not available	A 18-bed pediatric intensive care unit in a tertiary hospital	Ward	HCWs (medical officers)	SYS+FED	SYS (with AHR at point of care)	Direct observation by researcher and nursing staff	Before and after patient contact	Hand hygiene compliance (hand washing)	Australia
Dubbert (1990)	ITS	Total: 14 weeks<br>Baseline: 6 weeks<br>Intervention I: 4 weeks (Phrase I)<br>Intervention II: 4 weeks (Phrase II)	A 12-bed intensive care unit	Ward	Nurses	Phase I: EDU<br>Phase II: EDU+FED	None (unclear AHR use)	Direct observation by infection control	Procedure involved with patient contact	Hand hygiene compliance	USA

		Data on date is not available									
Mayer (2011)	Non-randomised trial	Total: 16 months CCT step-down<br>Baseline: 2 months<br>Intervention phase I: varied between 2 to 7 months<br><br>August 2000 to November 2001<br><br>ITS (less than 3 time points baseline);<br>2 years 9 months<br><br>April 2003 to December 2006	3 unit groups (6 wards) from a 450-bed hospital<br><br>Intervention 2 units (4 wards)<br>Control 1 unit (2 wards)<br>Step-down phase 1 and 2	Ward	HCWs	Phase I: SYS+EDU+FED<br>Phase II: WHO-5+INC	None (unclear AHR use)	Direct observation by trained part-time staff	Before, after, or before and after contact patient or patient's environment.	Hand hygiene compliance<br>Hospital acquired MRSA infection rate	USA
Benning (2011)	CBA	Total: 20 months (as a second phase of a national improving the quality of healthcare program)<br><br>March 2007 to September 2008	Total 18 hospitals<br><br>Intervention: 9 hospitals<br>Control: 9 matched hospitals<br>by size and geographic area	Hospital	HCWs	SYS+REM+SAF	None (with AHR)	n/a	Procurement	Soap and AHR consumption	England and Wales
Gould (1997)	CBA	Total: 3 months intervention period<br><br>Data on date is not available	Total 4 surgical wards from a teaching hospital<br><br>Intervention: 2 surgical wards<br>Control: 2 surgical wards	Ward/Individual	Nurses	EDU	None (unclear AHR use)	Direct observation by researchers	Frequency, appropriateness and duration of hand decontamination compliance and the use of	Hand decontamination compliance	UK

									gloves		

*SYS: system change, EDU: education, FED: feedback, REM: reminders, SAF: institutional safety climate, INC: incentives, GOAL: goal-setting, ACC: accountability, WHO-5: a combined intervention strategies including SYS, EDU,FED, REM, and SAF, AHR: alcohol based hand rub, HHC: hand hygiene compliance, HCW: healthcare worker, n/a: information is not available.
