# Supplementary material for: Comparative efficacy of interventions to promote hand hygiene in hospital: systematic review and network meta-analysis
Source: BMJ. 2015 Jul 28;351:h3728. doi: 10.1136/bmj.h3728 (PMC4517539; doi:10.1136/bmj.h3728)
Supplement: Supplementary file 7 [file luan024450.ww7_default.pdf]

Appendix 7: Funnel plots [posted as supplied by author]

Figure A
Contour-enhanced funnel plot of all ITS studies.

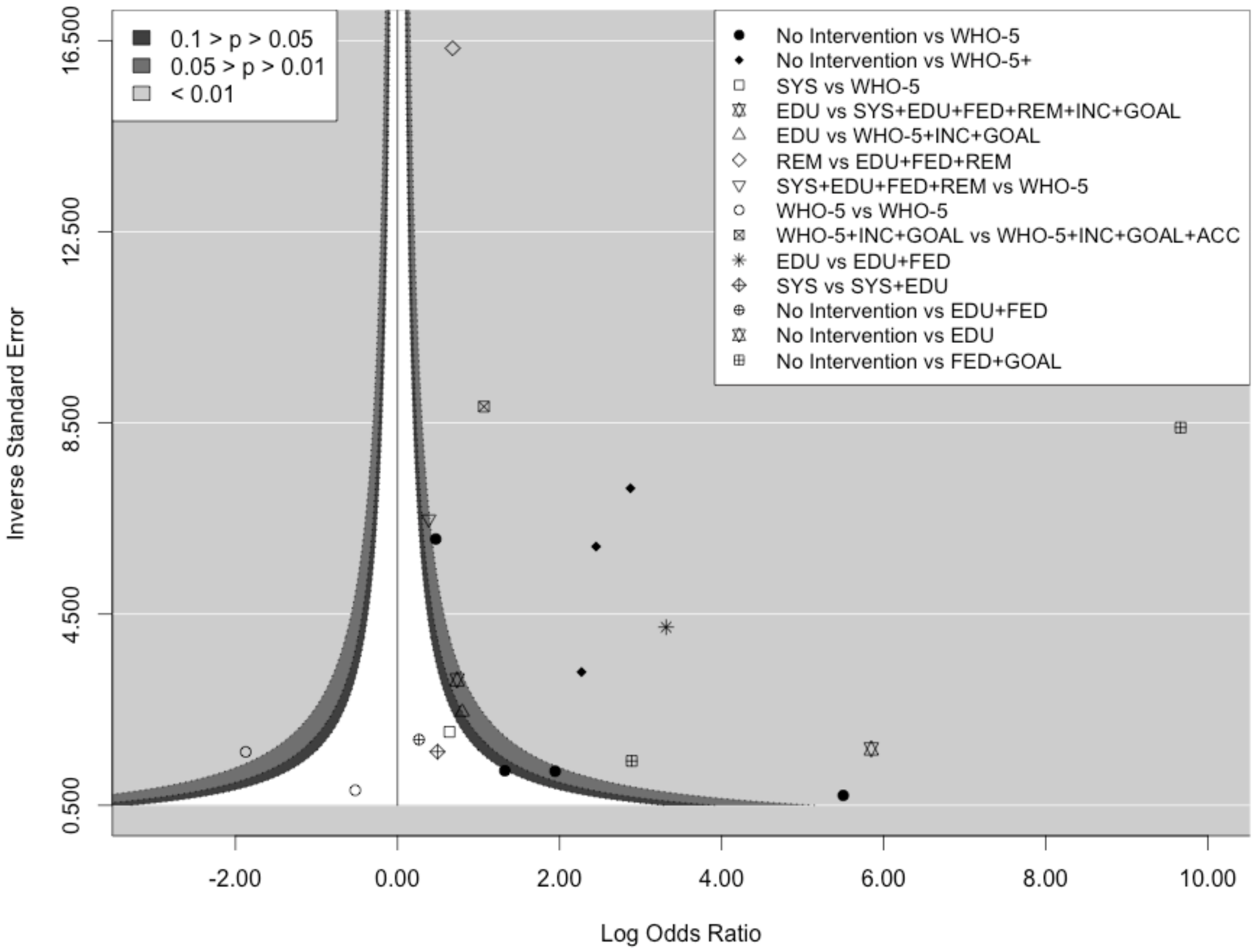

The contour-enhanced funnel plot of the effect size of all ITS studies. In this plot asymmetry with points selectively missing from the 0.1>p>0.05 region (black) and the p>0.1 region (white) would be suggestive of publication bias[1].

Evidence of such publication bias is not present here. However, interpretation is complicated by the fact that different studies are comparing different interventions so effect sizes might be expected to differ.

[1] Peters JL, Sutton AJ, Jones DR, Abrams KR, Rushton L (2008). Contour-enhanced meta-analysis funnel plots help distinguish publication bias from other causes of asymmetry. J Clin Epidemiol 61:991-6.

Figure B Contour-enhanced funnel of ITS studies comparing no intervention with WHO-5.

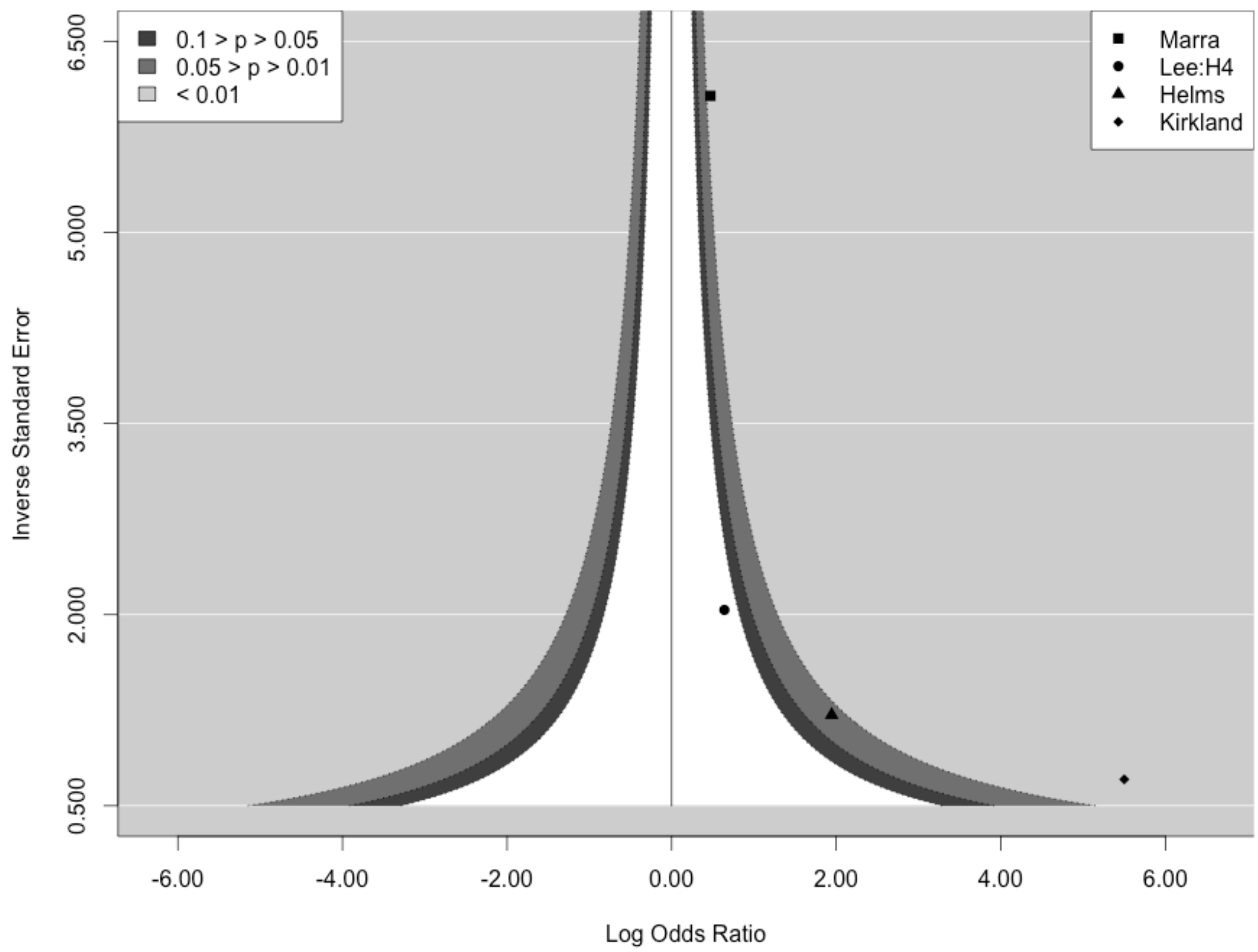

Figure C
Contour-enhanced funnel of ITS studies comparing no intervention with WHO-5+.

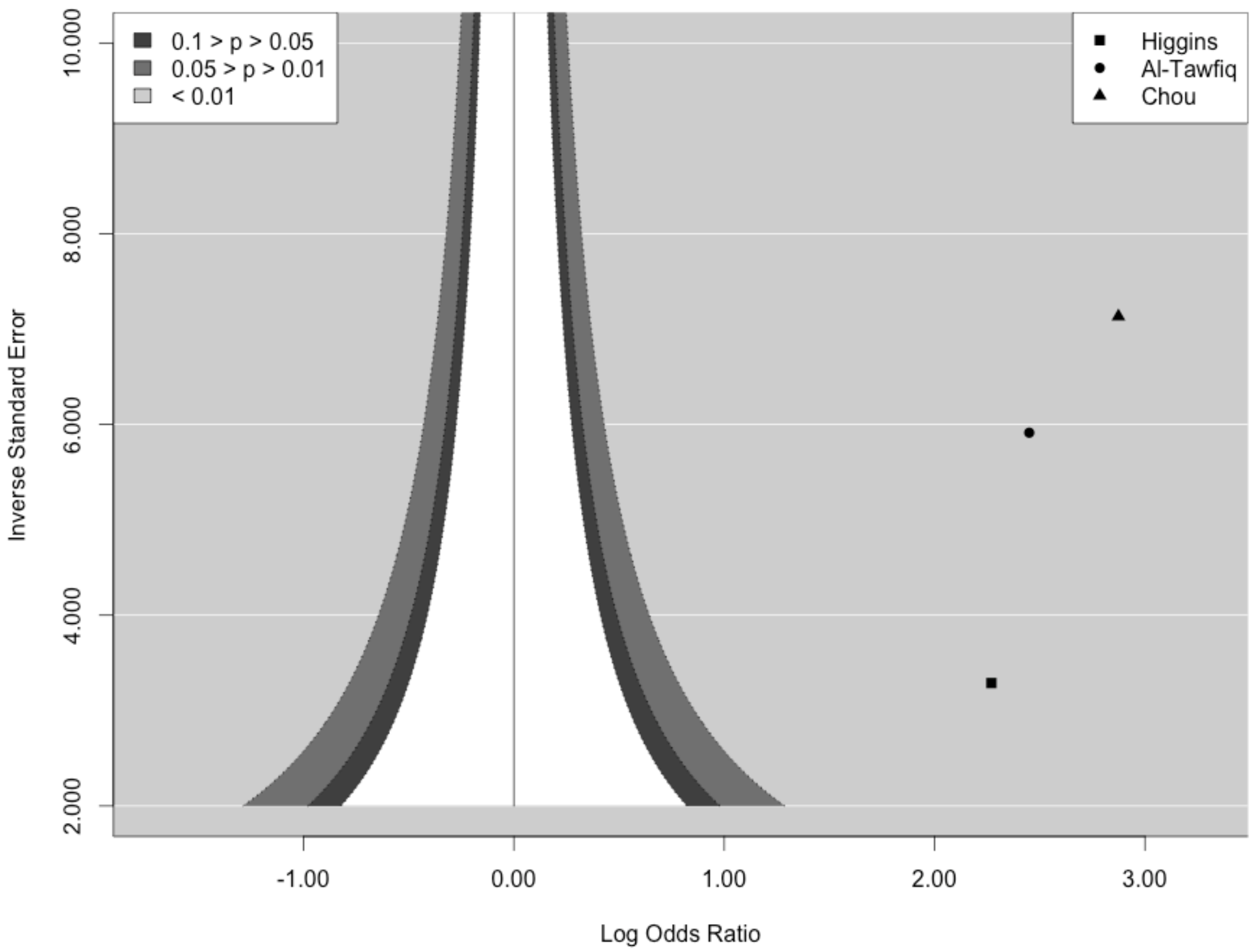

Figure D
Contour-enhanced funnel of RCTs comparing WHO-5 with WHO-5+.

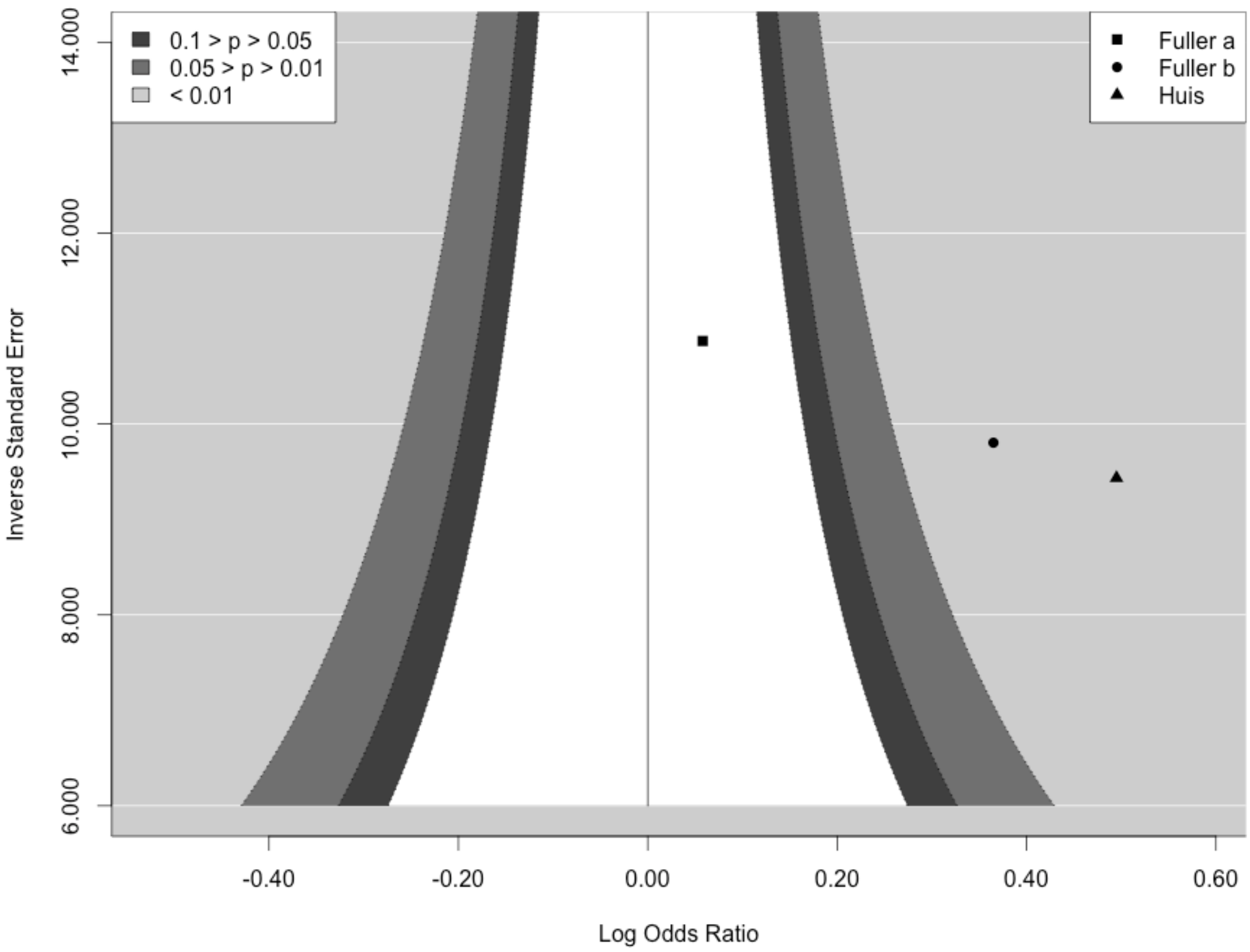

None of the above three contour-enhanced funnel plots of specific pair-wise comparisons provide any clear evidence of publication bias (which would have been indicated by studies "missing" from the areas where p>0.05 indicated by the white areas and the area of darkest shading).
