# Supplementary material for: Comparative efficacy of interventions to promote hand hygiene in hospital: systematic review and network meta-analysis
Source: BMJ. 2015 Jul 28;351:h3728. doi: 10.1136/bmj.h3728 (PMC4517539; doi:10.1136/bmj.h3728)
Supplement: Supplementary file 8 [file luan024450.ww8_default.pdf]

Appendix 8: Details of extracted intervention components and level of information on resource use. [posted as supplied by author]

Author (year)	Study design	1.System Change	2.Education & Training	3.Feedback	4.Reminders	5.Institutional safety climate	6.Others including Goal-setting, Incentives, and Accountability	Control/Baseline intervention	Level of information on resources use High (H), Medium (M) or Low (L)
Fuller (2012)	CRCT	Not done but available as part of the national campaign	Not done but available as part of the national campaign	Observation and feedback by "Ward Coordinator" performed on one repeating 4-week cycle. The tasks were hand hygiene observation of an individual health care worker, and immediate feedback as well as preparing an action plan to feed back at a ward meeting.<br>Training program for observers is required (Total 62 training visits, 1 to 1.5 hour)	Not done but available as part of the national campaign	Not done but available as part of the national campaign	Goal-setting: Ward coordinators were asked to fill out a form to record observations, feedback, goals and action plans.	National "Cleanyourhands" campaign as routine practice (similar to WHO-5)	M
Huis (2013)	CRCT	Adequate product and facilities	Education for improving relevant knowledge and skills.<br><br>Distribution of educational material/written information about hand hygiene<br><br>Website	Feedback<br>Bar charts of hand hygiene rates	Reminders<br>Distribution of posters replaced every 12 weeks<br>Interviews and messages in newsletters or hospital magazines<br>General reminders by opinion leaders/ward management	Gaining active commitment and initiative of ward manager.<br>Modelling by informal leaders at the ward; demonstrating good hand hygiene behavior, instructing and stimulating their colleagues	Goal-setting: Setting norms and targets within the team<br>Three interactive team sessions (1–1.5 hour each)<br>Analysis of barriers and facilitators to determine how nurses could best adapt their behaviour in order to reach their goal<br>Nurses address each other in case of undesirable hand hygiene behavior<br>All managers received a 4-hr training before the start of the intervention	State of the art strategy (SAS) implemented intervention 1 to 4 (System Change, Education and Training, Feedback, and Reminders)	H
Mertz (2010)	CRCT	Sink and AHR dispensers were available	Small group teaching seminars	Meeting of clinical manager and staff on the intervention units and the later meeting provide the specific performance feedback (biweekly meeting for 6 months)	Posters and pamphlets	Not done	Not done	System change was done<br>Sink and AHR dispensers were available before the intervention period in both control and intervention arm	M
Huang (2002)	CRCT	Not done	Educational intervention (Universal precaution training) provided by 3 trained investigator: including a) a 2-hr lecture, b) a 1-hr demonstration, and c) 30 min discussion	Not done	Not done	Not done	Not done	No intervention<br>but received training after the study finished	M
Fisher (2013)	RCT	AHR dispensers and basins were available at point of care	Not done	Quantified individual feedback by receiving confidential and weekly written feedback reports of hand hygiene compliance	Real-time reminders (audible beeps) using a wireless hand hygiene monitoring system	Not done	Not done	AHR dispensers and basins were available at point of care	M

Salamati (2013)	RCT	AHR dispensers and basins<br>were available at point of care	Hand hygiene education was performed by an infection control nurse via a 2 hour lecture; the lecture session was repeated a few times in such a way as to cover all the personnel working in different shifts	Motivational Interview; five sessions of interviewing with a maximum of 15 participants for 90 minutes	Not done	Not done	Not done	AHR dispensers were available at point of care<br>Hand hygiene education	M
Derde (2014)	ITS	Not done	Education sessions	Direct feedback after observation and monthly feedback of local compliance rates provided to wards to guide the content of each local hand hygiene program	Visual reminders (no details)	Not done	Not done	Reminder as posters	L
Lee (2013)	ITS	AHR at point of care	Training and education of healthcare worker	Observation and feedback of hand hygiene practices	Reminders in the workplace (e.g. posters)	Improving the safety climate in the institution with management support for the initiative	Not done	One unit was no intervention and another unit was system change changing AHR formulation. The other two were WHO-5.	L
Marra (2013)	ITS	Positive deviance (PD)* group changed the position of AHR dispensers and added AHR dispensers in the corridors<br><br>*PDs were defined as those HCWs who wanted to change, to think, to develop new ideas for improving HH and who stimulated other HCWs	Positive Deviants (PD) meeting with all HCWs twice monthly<br>The hospital PD coordinators provided PD training for all HCWs including nurses, physicians, physical therapists, speech pathologists, and nutritionists who used the dispensers and provided the opportunities to express feelings about hand hygiene.	PDs showed the HHC% and discussed their performance in every meeting	Some ideas and strategies were related to the reminders such as preparing badges for doctors who perform HH and noting them as examples and preparing a short theater presentation discussing "My 5 Moments for Hand Hygiene" with their peers	PD initiated engaging people to involve by inviting another PD in the next meeting	Not done	No intervention<br>(but AHR was available)	M
Al-Tawfiq (2013)	ITS	Increase availability of hand sanitizers (AHR)	Education<br>Formation of hand hygiene compliance team<br>Educational presentations	Feedback<br>Posting data on intranet<br>Compliance criteria shared with health care professionals<br>Inclusion in dashboard with goal-setting<br>Devotion of activity to low performing units<br>Face-to-face feedback during weekly tracer rounds<br>Frequent audit and feedback and discussed the feedback findings with each unit supervisor and fostering ways to improve	Promotion<br>Flashing pins "Wash your hands stay healthy"<br>Ask me "have you washed your hands" pins<br>Hand hygiene banners throughout the organization<br>Magnetic door posters promoting hand hygiene	Leadership commitment<br>Senior leadership engagement included monthly tracking of the compliance rates and communicating to management and hospital staff during monthly meeting and through the dashboard	Goal-setting:<br>Setting compliance goals<br>Increased the stated goal to 75%<br>Increased the goal to 85%	No intervention<br>(but AHR was available)	M
Armellino (2013)	ITS	Not done	Not done	Feedback metrics tabulated by a central server database delivered back to the HCWs through electronic light emitting diode boards, electronic mail summaries, and weekly performance reports	Not done	Not done	24 video cameras and motion sensors at handwashing sinks and sanitizer dispensers to record hand hygiene opportunities<br>Goal-setting: Setting the targeted compliance as >=95%	Video cameras were installed during baseline period as well but without feedback	M

Armellino (2012)	ITS	Not done	Not done	Feedback metrics tabulated by a central server database delivered back to the HCWs through electronic light emitting diode boards, electronic mail summaries, and weekly performance reports	Not done	Not done	24 video cameras and motion sensors at hand washing sinks and sanitizer dispensers to record hand hygiene opportunities<br>Goal-setting: Setting the targeted compliance as >=95%	Video cameras were installed during baseline period as well but without feedback	M
Chan (2013)	ITS	38 AHR dispensers were installed and changed the location	Not done	Not done	Not done	Not done	Not done	No intervention<br>(but the AHR was available)	M
Crews (2013)	ITS	More than 900 wall dispensers were installed and substituted with new alcohol-based hand rubs.	Annual educational training for clinical staff including physician and hospital staff	Routine feedback to HCWs	Marketing committee launched a campaign that emphasized branding hand hygiene with a positive image<br><br>Slogan and child-friendly posters and signs with the message were displayed at strategic locations<br><br>Additional items containing the message, including pens, buttons, calendars, and coloring books, were widely distributed	Not done	Goal-setting: Hand hygiene goal added to employee<br>Three goals related to quality or patient safety<br><br>Reward incentives: If the goals are achieved, every employee receives a financial reward	Multiple unit-based educational<br>initiatives and use of a gel-based alcohol hand rub	M
Salmon (2013)	ITS	Not done	Hand hygiene auditor training program based on the WHO "My 5 moments for hand hygiene" in the orientation program for final year nursing students,<br>1- hour session including lecture and practical auditing using WHO video tools and 398 nursing students from 3 nursing schools involved	Not done	Not done	Not done	Not done	No intervention/routine practice	M

Talbot (2013)	ITS; Phase I	As part of the bundle of readiness assessment and planning program	Expanded HH direct observation program<br><br>Observation program was expanded to include all inpatient and outpatient locations<br><br>The observers attended required training on a standardized observation methodology	Readiness assessment and planning<br><br>The project bundle focused planners on addressing the following: defining the problem, ensuring project alignment with the organization's mission, securing financial support, defining performance and measurement objectives, and establishing leadership commitment	System-wide marketing campaign<br><br>Poster messaging and targeted talks aimed to increase HH awareness and its importance in preventing HAIs	Leadership goal-setting<br>Improved HH adherence was adopted as an institutional quality improvement goal and the performance related to the goal immediately became a factor in annual performance evaluations and incentive compensation for medical center leaders.	Goal-setting:<br>Modest HH adherence goals were set in the first year of the program (adherence of 65% as a threshold goal, 75% as a target goal, and 85% as a reach goal) with the intent of increasing performance requirements each year<br><br>Reward incentive:<br>Financial incentives via a self-insurance trust allocation rebate program. The component of the allocation rebate was worth up to 25% of the total rebate dollars (2.5% of yearly premiums). For example, for a physician whose yearly premium was $10,000.00, the rebate amounted to $250.00.	Phase I: Hand hygiene annual faculty and staff training	M
	Phase II	Same as Phase I above	Same as Phase I above	Same as Phase I above	Same as Phase I above	Hand Hygiene executive committee<br><br>The committee consisted of key physician and nursing leaders and was established to review location performance monthly and direct interventions	Goal-setting and Reward incentive<br>Same as Phase II above, a follow-up campaign<br><br>Location-specific accountability interventions<br><br>Units with low adherence were identified for interventions on the basis of a system-wide hand hygiene intervention pyramid<br><br>Structured individual accountability interventions<br>Observers provided direct feedback when a hand hygiene opportunity was missed. System leadership monitored event reporting and acted as necessary, consistent with organizational policies concerning behaviors that undermine a culture of safety	Phase II: Intervention Implemented in Phase I	

Higgins (2013)	ITS	Increased supplies of hand AHR, dispenser at bedside	Adenosine triphosphate (ATP) monitoring system, a mobile stand-alone computer system, was purchased and used in the clinical area during spot audits and also at regular intervals outside the staff canteen to measure handwashing technique<br><br>HCWs were selected at random and asked to wash their hands with soap and water. Once the hands were completely dry, the swab was rubbed against the tips of each finger, in between each finger and then in an S-shape along the palm of one hand	Monthly hand hygiene audit and verbal feedback provided directly to staff during the audits.	Posters displaying hand hygiene technique and information of "WHO 5 moments" placed at the key area of the hospital.<br><br>Advertising campaign was carried out in the hospital through e-mails and general hospital mail. An information leaflet was designed and copies were left in the canteen, at nurses' stations, in staff meeting rooms etc.	Commitment from management, hand hygiene audit results were provided to the hospital executive team and board	Reward incentive:<br>Fob watches were provided as spot prizes	No intervention<br>(but the AHR was available)	M
Helder (2012)	ITS	Not done	Not done	Not done	Screen servers for 6 computer screens, 2 per unit, were involved to emphasize the need for improved adherence to hand hygiene protocols and were designed according to theoretical principles of message framing. The messages on the screen servers were replaced by a newly designed 2-screen series every 2 weeks	Not done	Not done	AHR dispensers were available at point of care. However, five months before the study present, a multidisciplinary infection prevention education program was organized	M
Kirkland (2012)	ITS	Optimised availability of hand sanitiser	Education and training<br><br>Developed electronic learning module and training video that provided hand hygiene education for all staff. It was accessible through the hospital intranet. A 'certification' program was also available by which staff demonstrated HH competency	Measurement and feedback<br>Routine HH audits<br>Monthly unit specific data published on an intranet site available to all staff, and reported to executive leadership, clinical leaders and board members	Marketing and communication<br><br>Marketing staff created a series of posters and screen savers, stories in medical center publications and local news outlets, and direct communication with staff about expectations and progress towards goals	Leadership and accountability<br><br>Leadership publicly emphasised the importance of hand hygiene	Not done	No intervention<br>(but AHR was available)	M

Morgan (2012)	ITS	Installed multiple automated, networked, touch free AHR and soap dispensing units at the entrance to each room and the sink in each room	Infection control and research staff monthly visited each unit to present the poster, remind staff about the importance of hand hygiene, and answer any questions about the study<br><br>The WHO 5 Moments for Hand Hygiene were discussed in training.	Feedback compliance was provided for entry and exit based on human observation	Two posters in each unit to display unit-specific monthly and quarterly hand hygiene compliance rate also included infection control reminders to link hand hygiene with infection prevention (e.g. unit infection rates, photos of unit staff performing hand hygiene, general HAI education)	Not done	Not done	No intervention<br>(but the AHR was available)	M
Stone (2012)	ITS	Alcohol hand rub at bedside	Not done	Regular audit and feedback of compliance	Posters reminding HCWs to clean their hands	Empowering patients to remind HCWs	Not done	No intervention/routine practice	L
Jaggi (2012)	ITS	Not clear	Not clear	Not clear	Not clear	Not clear	Identify key area of improvement<br><br>Training for a bundle of prevention<br><br>Auditing	No intervention/routine practice	L
Lee (2012)	ITS	Dispenser installation and pocket-sized containers provided	All HCWs received continuing education and pre-service education on hand hygiene issues by experienced infection control nurses (ICN)	Monitoring and feedback of hand hygiene compliance monthly by infection control nurses	Posters	Not done	HCWs were encouraged to educate their patients and families about proper hand hygiene.	No intervention/routine practice	L
Mestre (2012)	ITS	AHRs were placed at all bedsides on high risk areas (ER, ICUs)	Theoretical and practical workshop to all HCWs and practical sessions	Audit by a hand hygiene monitor team<br>8 HCWs direct observation with 2 evaluation periods and 25 days of monitoring<br><br>Feedback through informal interactive session on every ward at the end of evaluation periods (2 sessions)	Posters and handout, replaced monthly	Commitment by administrative and nursing director	Not done	Phase I: No intervention/routine practice<br><br>Promotion of hand hygiene such as staff education, reminders, and six months hand hygiene audit was performed during baseline period but it was neither structured nor sustained on time.	H
		AHRs were placed at all bedsides in conventional wards while maintaining those at corridors	Maintain as above	Audit by a hand hygiene monitor team<br>8 HCWs carried out direct observation with 17 evaluation periods and 51 days of monitoring.<br>3 randomized days every 3 weeks ("3/3 strategy")<br><br>Feedback using control charts on every ward at institutional and individual level	Maintain as above	Maintain as above<br><br>Corrective actions: Modification of incorrect HH habits, clarification of doubts and positive reinforcement were conducted	Not done	Phase II: Intervention Implemented in Phase I.	

Koff (2011)	ITS	Not done	A personalized hand hygiene device was worn by HCWs used for recording the frequency of hand disinfection event	Feedback was provided to both individuals and the entire group	Not done	Not done	Not done	Wall-mounted dispensers were installed	M
Doron (2011)	ITS	Hand-sanitizer dispensers provided in all public non-patient care areas	Educational program; online teaching, grand rounds lectures and nurses	Close observation with feedback	Promoting campaign by email to introduce the campaign to employees<br>Posters; large size to introduce to patients and families, small size for the walls in various places)<br><br>Handout for new patients<br>Stickers and pins with positive and humorous messages<br>A private advertising firm was contracted to develop a professional marketing campaign for the hospital	Leadership commitment<br><br>Chief medical officer and CEO spoke about HH at every given opportunity<br><br>Heads of department and ICU directors were asked to make hand hygiene an educational priority and to personally carry out hand hygiene observation	Not done	During 2007 to July 2008 (baseline period), intervention component 1 to 4, including placement of hand sanitizers, reminder signs, education, and feedback with observed compliance, was implemented but reinforced with a new strategy together with component 5 during the intervention period.	M
Marra (2010, 2011)	ITS	Changing the position of alcohol gel dispensers in the patient rooms and to put more in the corridors	Positive deviants (PD) meeting with all SDU HCWs twice monthly, 1.5 hour each, attendance about 35-40 to discuss and provide training for all HCWs including nurses, physicians, physical therapists, speech pathologists, and nutritionists who used the dispensers and provide the opportunities to express feelings about hand hygiene	PD showed the hand hygiene compliance and discussed their performance in every meeting	Incorporated laminated sheets on "My Five Moments for Hand Hygiene" as the first page in all of SDU patient medical records	PD initiated engaging people to involve by inviting another PD in the next meeting	Not done	No intervention (but the AHR was available)	M
Yngstrom (2011)	ITS	Alcohol hand disinfection at every bed together with pictures and posters, and instruction	Meeting monthly (reporting, evaluation, feedback and discussion) and continuous education program (Department level)	Feedback of hand hygiene performance during the meeting	Not done	Not done	Goal-setting:<br>The goal was a 40% reduction in healthcare associated infections in ventilated patients<br><br>Process objective<br><br>The process objective was 100% of staff to implement basic hygiene routines	No intervention (but the AHR was available)	L

Helms (2010)	ITS	Alcohol foam dispenser installed both inside and outside patients room.<br><br>Pocket-sized container for all staff<br><br>Hand sanitizing station in the main lobby, emergency lobby and waiting rooms	Aggressive education program<br><br>Implementation of "You bugged me" program, staff member presenting another employee with a card if they witnessed them not washing their hand properly<br><br>The infection control coordinator attended all the staff meeting in all departments and provided educations on proper hand hygiene technique<br><br>One part of the program is the use of the fluorescent lotion to see effects of handwashing	Direct feedback when staff forget to perform hand hygiene via "You bugged me" program	Signs to remind the staff to wash their hands.<br><br>Flyers to educate patients' visitors<br><br>The patients were educated on admission to remind the staff to wash their hands.	Chief executive Officer (CEO) and Chief Nurse Officer (CNO) involved in activity for the penalty of non compliance	Not done	No intervention (but the AHR was available)	M
Chou (2010)	ITS	Adding AHR dispensers in each patient room<br><br>Installed in all public area including outpatient clinics	Enhanced educational material includes added interactive demonstration "fluorescent germs" and bacterial cultures of hands before and after hand hygiene to hospital-wide educational programs to impress the important of hand hygiene and posters contest	Hand hygiene liaison (at least one staff each department) responsible for review in HH policy, ensuring availability of HH product, observing HH at least 20 opp. each month)<br><br>Feedback in hand hygiene compliance	Posters from the contest displayed in the key areas	Hospital wide support; the bundle of this intervention was introduced to hospital administration for their support and approval and presented to multiple leadership committees consisting of physicians, nursing directors and managers and other leaders<br><br>A violation letter was sent to managerial personnel of noncompliant individuals to take corrective action with violators.	Goal-setting and Reward incentive:<br>Nursing units were rewarded with pizza parties if they achieved and sustained the targeted hand hygiene compliance	No intervention (but the AHR was available)	M
Vernaz (2008)	ITS	Pocket-sized containers using AHR were available during baseline and intervention)	Not clear	Not clear	Not clear	Not clear	Spring 2003: Applying social marketing theory to promote standard precautions and isolation precautions mentioned hand hygiene as an element of standard precautions (did not target the promotion of AHR in particular)<br><br>Autumn 2005: Swiss national hand hygiene promotion campaign and the global patient safety challenge entitled 'Clean your hand is safer care' with an exclusive focus on the frequent and proper of AHR	Pocket-sized bottles for AHR were provided	L

Whitby (2008)	ITS<br>1) Washingtoncampaign	AHR placed at the end of each bed, chart trolleys and in medication preparation areas<br><br>Liquid soap provided at handwashing basin<br>(Pre intervention: 1 month)	Pre intervention: 4 months<br>A series of meetings led by seniors and attended by all clinical and non-clinical staff<br><br>Intervention phase 1: 2 months<br>Informal lectures by the project nurses	Intervention phase 1: 2 months<br>Staff developed talking-wall promotional cartoons with prizes awarded and the additional cartoons developed by an external artist	Intervention phase 1: 2 months<br>Information in accordance with CDC's guidelines was provided via pay slips	Intervention phase 2: 3 months<br>"Walk-arounds" by executive medical and nursing members and photograph of senior staff with speech balloons at each ward in the last month	Large photographs of the hospital executive were positioned throughout the wards	No intervention (but the AHR was available)	M
	2) Geneva campaign	AHR placed at the end of each bed, chart trolleys and in medication preparation areas<br><br>Liquid soap provided at handwashing basin.<br>(Pre intervention: 1 month)	Pre intervention: 5 months<br>Clinician-led meetings, semi-structured format all clinical and non-clinical staff	Not done	Intervention phase 1: 2 months<br>Posters and Screen savers	Intervention phase 2: 5 months<br>"Walk-arounds" by executive medical and nursing members AND Photograph of senior staff with speech balloons at each ward in the last month	Not done	No intervention (but the AHR was available)	
	3) AHR substitution	AHR placed at the end of each bed, chart trolleys and in medication preparation areas.<br>Liquid soap provided at handwashing basin<br><br>(Pre intervention: 1 month) (Pre intervention: 4-5 months)	Not done	Not done	Not done	Not done	Not done	No intervention (but the AHR was available)	
Grayson (2008)	ITS	Provide a supportive environment to encourage and promote AHR use by provide the ready access to AHR and access to moisturiser	Provide education to enable staff to learn correct hand hygiene	Monitoring and feedback of hand hygiene compliance to provide encouragement	Increase awareness of the importance of hand hygiene including promotional initiatives	Provide a supportive environment to encourage hand hygiene compliance with strong executive and clinical leadership	Not done	No intervention/routine practice	M
Eldrige (2006)	ITS	Provide AHR container at point of care, pocket-sized container to all staff, antimicrobial soap and hand lotion	Update annual infection control training to be consistent with CDC Guideline	Monitor HCWs' adherence to hand hygiene practices. Provide information regarding the workers' performance.	Posters were posted in staff-only areas, patient-care areas and waiting areas with monthly rotation.<br><br>Promote "It's Okay to Ask" attitude: Caregivers, visitors, and patient should feel free to ask caregivers if they have cleaned their hands	Make improved hand hygiene an institutional priority and provide administrative and financial support	Not done	No intervention/routine practice	L

Johnson (2005)	ITS	Provide a supportive environment to encourage and promote AHR use by provide the ready access to AHR and access to moisturiser	Provide education to enable staff to learn correct hand hygiene	Monitoring and feedback of hand hygiene compliance to provide encouragement.	Increase awareness of the importance of hand hygiene including promotional initiatives	Provide a supportive environment to encourage hand hygiene compliance with strong executive and clinical leadership	Not done	No intervention/routine practice	M
Khatib (1999)	ITS	Not done	All received in-service instructions on appropriate hand-washing techniques and the use of gloves when caring for mechanically ventilated patients consisted of formal lectures and demonstrations	Not done	Wash Hands Use Gloves labels were permanently placed on all ventilators to remind the policy of hand washing and gloves use without feedback regarding to the outcome	Not done	Not done	Education	L
Tibballs (1996)	ITS	Newly contructed ICU.<br>Hand washing basins are located in each room within two to three metres of each bed and in the corridor	Not done	Performance feedback on hand hygiene compliance rate display were placed in strategic location in the ICU	Not done	Not done	Not done	System change	L
Dubbert (1990)	ITS	Not done	Four 15-minute classes taught within a one-week period by ICN and scheduled to include all tours of duty and all of nursing staff	Group feedback intervention<br><br>The observers posted a feedback about the percentage of hand washing "error" and specific information about the nature of any errors involving critical procedures but did not identify individual subjects	Not done	Not done	Not done	No intervention/routine practice	L
Mayer (2011)	Non-randomised trial	Positioning dispensers of alcohol sanitizer in convenient locations	Education program providing standardized unit in-service presentations prepared by infection preventionist, the hospital epidemiologist, physician groups, and infection control personnel and clinical staff.	Ongoing audit with monthly feedback by infection preventionist	Not done	Not done	Not done	No intervention/routine practice	H

	ITS	Introduce AHR and positioning the dispensers	Education program providing standardized unit in-service presentations prepared by infection preventionist, the hospital epidemiologist, physician groups, and infection control personnel and clinical staff	Ongoing audit with monthly feedback by infection preventionist	Posters with catchy phrases were placed throughout the hospital	Monthly meeting of a hand hygiene committee comprising infection preventionists, nurse managers, service directors and hospital epidemiologist to encourage staff involvement and to provide unit specific feedback	Reward incentive: through "Positive reinforcement"<br><br>The hand hygiene committee generated new motivational campaign themes to maintain interest<br><br>An example of a group motivator theme was the "War on Germs" to encourage unit teamwork<br><br>Publicizing that the unit with the best hand hygiene compliance would win a pizza party Individual incentives theme, in which individuals who were caught in the act of performing hand hygiene were entered into monthly drawings to win prizes.	No intervention/routine practice	
Benning (2011)	CBA	Make AHR available at the bedside	Not done	Not done	Posters on wards updated monthly	Encouraged patients to ask staff to clean their hands	Not done	Control: no intervention but "cleanyourhands" campaign implemented during 2004-2005 (in both groups)	L
Gould and Chamberlain (1997)	CBA	Not done	Educational program by experienced nurse teachers with specific expertise (5 different sessions, 30 min each)	Not done	Not done	Not done	Not done	No intervention/routine practice	M

* AHR: alcohol based hand rub, CRCT: cluster randomised controlled trial, RCT: randomised controlled trial, ITS: Interrupted time series study, CBA: controlled before and after study.
