# Supplementary material for: Comparative efficacy of interventions to promote hand hygiene in hospital: systematic review and network meta-analysis
Source: BMJ. 2015 Jul 28;351:h3728. doi: 10.1136/bmj.h3728 (PMC4517539; doi:10.1136/bmj.h3728)

Appendix 9: Supplementary results [posted as supplied by author]

Supplementary Figure 1. Box-and-whiskers plot showing relative efficacy of different hand hygiene intervention strategies compared with standard of care when excluding studies where interventions were made at multiple time points from the network meta-analysis of interrupted time series studies. Lower and upper edges represent 25th and 75th percentiles from the posterior distribution; the central line represents the median. Whiskers extend to the 5th and 95th percentiles. Intervention strategies were as follows: single intervention; WHO-5; and WHO-5+ (WHO-5 combined with either Incentives, Goal-setting, or Accountability).

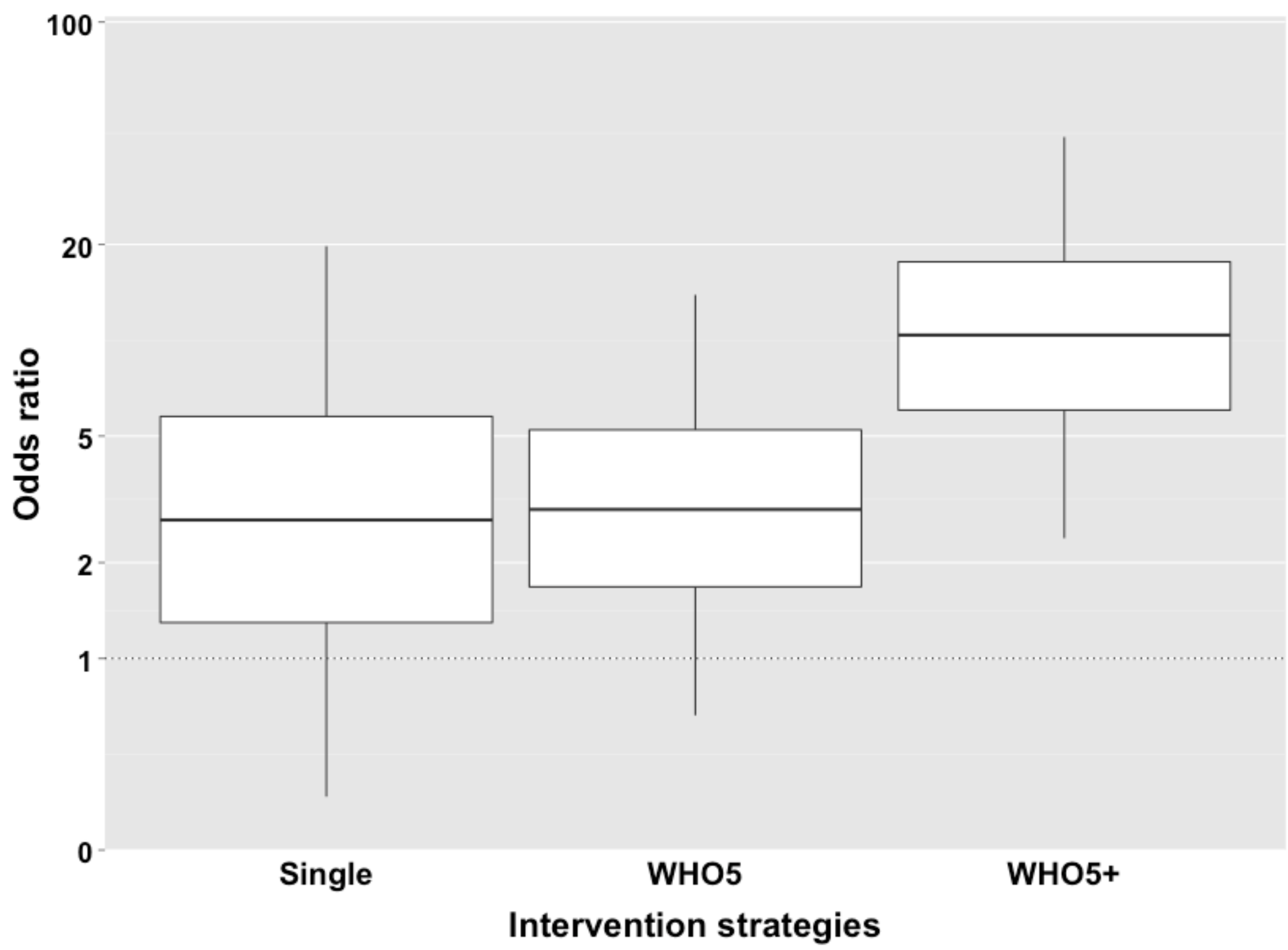

Supplement: Supplementary file 9 [file luan024450.ww9_default.pdf]
